# Development of high-voltage and high-energy membrane-free nonaqueous lithium-based organic redox flow batteries

**DOI:** 10.1038/s41467-023-40374-y

**Published:** 2023-08-08

**Authors:** Rajeev K. Gautam, Xiao Wang, Amir Lashgari, Soumalya Sinha, Jack McGrath, Rabin Siwakoti, Jianbing “Jimmy” Jiang

**Affiliations:** Department of Chemistry, University of Cincinnati, P.O. Box 210172, Cincinnati, OH 45221 USA

**Keywords:** Batteries, Batteries, Batteries

## Abstract

Lithium-based nonaqueous redox flow batteries (LRFBs) are alternative systems to conventional aqueous redox flow batteries because of their higher operating voltage and theoretical energy density. However, the use of ion-selective membranes limits the large-scale applicability of LRFBs. Here, we report high-voltage membrane-free LRFBs based on an all-organic biphasic system that uses Li metal anode and 2,4,6-tri-(1-cyclohexyloxy-4-imino-2,2,6,6-tetramethylpiperidine)-1,3,5-triazine (Tri-TEMPO), N-propyl phenothiazine (C3-PTZ), and tris(dialkylamino)cyclopropenium (CP) cathodes. Under static conditions, the Li||Tri-TEMPO, Li||C3-PTZ, and Li||CP batteries with 0.5 M redox-active material deliver capacity retentions of 98%, 98%, and 92%, respectively, for 100 cycles over ~55 days at the current density of 1 mA/cm^2^ and a temperature of 27 °C. Moreover, the Li||Tri-TEMPO (0.5 M) flow battery delivers an initial average cell discharge voltage of 3.45 V and an energy density of ~33 Wh/L. This flow battery also demonstrates 81% of capacity for 100 cycles over ~45 days with average Coulombic efficiency of 96% and energy efficiency of 82% at the current density of 1.5 mA/cm^2^ and at a temperature of 27 °C.

## Abstract (web summary)

Redox flow batteries are promising energy storage systems but are limited in part due to high cost and low availability of membrane separators. Here, authors develop a membrane-free, nonaqueous 3.5 V all-organic lithium-based battery and demonstrate its operation in both static and flow conditions.

## Introduction

Large-scale electrical energy storage (EES) systems are vital for the efficient utilization of widely available intermittent renewable energy sources such as solar and wind energy to mitigate the mismatch between the generation and consumption of electrical energy^1^. Rechargeable batteries are the first choice for building advanced EES systems owing to their high efficiency and flexible installation. Among the various battery technologies being explored, redox-flow batteries (RFBs) have attracted particular attention as promising EES systems because of their unique feature of the decoupling of energy density and power^2,3^. Based on the electrolyte used, RFBs can generally be classified into two types: aqueous and nonaqueous RFBs^4,5^. Aqueous RFBs use water as the solvent for the anolyte and catholyte, as this offers several advantages, including fast reaction kinetics, low cost, nonflammability, and high ionic conductivity^6^. Remarkable progress has been made with regard to aqueous RFBs, and they have been commercialized^7,8^. However, aqueous RFBs suffer from two major problems: limited cell voltage owing to the narrow electrochemical window of water (1.23 V) and low energy density (20–50 Wh/L)^6,9^. In contrast, nonaqueous RFBs (NRFBs) exhibit a wider electrochemical window (up to 6 V) and potentially higher energy density^10,11^. Moreover, the fact that one can employ widely available solvents and supporting electrolytes allows for the use of a range of redox-active compounds^12,13^. However, the development of NRFBs has been hindered by the limited availability of critically important ion-selective membranes, the low ionic conductivity of nonaqueous electrolytes, and the high costs of these materials^12,14^. Specifically, the limited availability of appropriate ion-selective membranes and their high cost (approaching 40% of the total battery cost) are the key challenges^15^. To address these limitations, cost-effective size-exclusive separators have been used in NRFBs as alternatives to ion-selective membranes^16,17^. These porous-separator-based batteries can exhibit high current densities; however, this strategy has several downsides, including low rates of active material utilization, low Coulombic efficiency, and self-discharging^18^.

Thus, several membrane-free batteries have been proposed and developed. Initially, classical fluid dynamics engineering based on the laminar flow of electrolytes through parallel microchannels was exploited to develop membrane-free batteries. However, these microfluidic batteries can only be realized at the microscale and thus are not suitable for large-scale energy storage^19,20^. Recently, immiscible electrolyte-based liquid–liquid biphasic systems have received significant attention for the construction of membrane-free batteries. The liquid–liquid interface of these biphasic systems separates the catholyte and anolyte and functions as a natural barrier, thus eliminating the need for a membrane. Unlike the case for laminar-flow batteries, the biphasic membrane-free approach allows for the design of flow batteries with higher power and capacity. Recently, several aqueous biphasic systems (ABSs) (aqueous/nonaqueous phases) were reported in the literature^19,21–27^. However, most of the reported membrane-free ABSs were investigated under static conditions and showed limited scalability^28–30^. Membrane-free batteries have rarely been investigated under actual flow conditions because of the convective-mass-transport-induced disturbances at the liquid–liquid interface under flow conditions, which results in self-discharging and active material crossover^17,31^. Moreover, ABS batteries typically show low energy density owing to their limited cell voltage^32,33^. Therefore, membrane-free batteries based on nonaqueous electrolytes have been developed to increase the energy density^12,34^. However, the development of flow batteries based on a nonaqueous biphasic system (NBS) has been hindered by the lack of immiscible organic solvents and redox-active materials that exhibit suitable solubilities in each phase to prevent active material crossover in the self-stratified biphasic system. Liu et al. demonstrated a static membrane-free battery-based all-organic NBS using Li metal in nonafluoro-1,1,2,2-tetrahydrohexyl-trimethoxysilane (NFTOS) as the anolyte and 2-ethylanthraquinone (2-EAQ) and benzo[1,2-b:4,5-b’]dithiophene-4,8-dione (BDTD) tetraethylene glycol dimethyl ether paired together as the catholyte^23^. Both catholyte materials (i.e., 2-EAQ and BDTD) exhibit low solubilities in the anolyte (NFTOS), thus allowing for the study of the charge/discharge cycling characteristics. However, this battery suffers from (1) low charge/discharge current density (50 µA/cm^2^), (2) relatively low nominal cell voltage (~2.2 V) for both cathodic materials and (3) a lack of information regarding its capacity retention rate and cycling characteristics under flow conditions^23^. Hence, there is an urgent need to develop membrane-free batteries that use flowable nonaqueous electrolytes with high voltage and energy density.

**Fig. 1:**
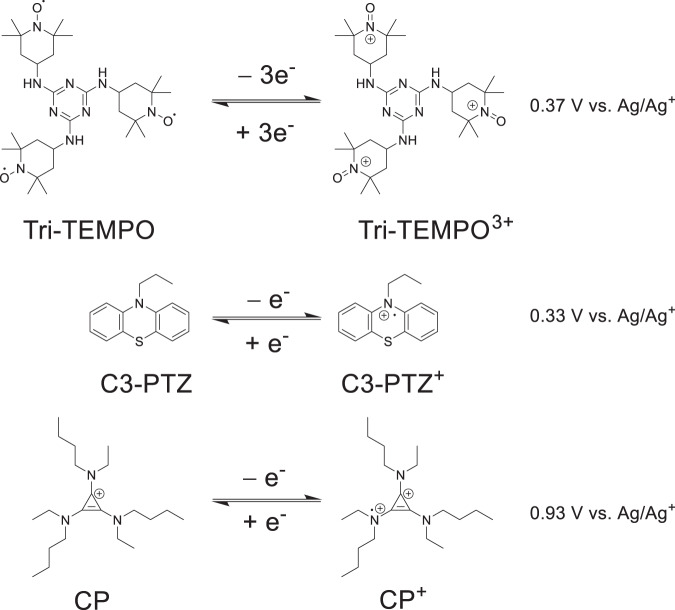
Chemical formulas and redox voltages of organic redox materials. Electrochemical reactions of Tri-TEMPO, C3-PTZ, and CP: Understanding the Redox Mechanisms and Charge Transfer Processes.

In this work, we report an all-nonaqueous biphasic membrane-free battery that shows high voltage and energy density under both static and flow conditions. The NBS-based battery employs Li metal in an ionic liquid (1-butyl-1-methylpyrrolidinium bis(trifluoromethylsulfonyl)imide (BMP-TFSI)) as the anode electrolyte solvent (top phase) and a set of metal-free organic compounds (Fig. 1), which include TEMPO derivative (2,4,6-tri-(1-cyclohexyloxy-4-imino-2,2,6,6-tetramethylpiperidine)-1,3,5-triazine (Tri-TEMPO)), phenothiazine derivative (N-propyl phenothiazine (C3-PTZ)), or cyclopropenium derivative (tris(dialkylamino)cyclopropenium (CP)) in fluoroethylene carbonate (FEC) as the cathode electrolyte solvent (bottom phase). The Li||Tri-TEMPO, Li||C3-PTZ, and Li||CP biphasic batteries exhibited theoretical cell voltages of 3.53, 3.49, and 4.09 V, respectively. The developed biphasic system was investigated under both static and flow conditions using the catholyte in different concentrations (0.1, 0.2, and 0.5 M). The static Li||Tri-TEMPO, Li||C3-PTZ, and Li||CP batteries with 0.5 M catholyte displayed capacity retention rates of 98%, 98%, and 92%, respectively, after 100 cycles over ~55 days at a current density of 1 mA/cm^2^ and a temperature of 27 °C. Moreover, under flow conditions, the prolonged cycling of the 0.5 M Li||Tri-TEMPO battery resulted in a capacity retention rate of 81% after 100 cycles over ~45 days. The cycling was conducted at a higher current density of 1.5 mA/cm² and a temperature of 27 °C. The Coulombic efficiency (CE), voltage efficiency (VE), and energy efficiency (EE) were measured to be 96%, 85%, and 82%, respectively. The flow battery exhibits a high cell voltage of 3.53 V, resulting in a high energy density of approximately 33 Wh/L. Pre- and post-cycling battery analysis confirmed the absence of crossover of the active materials.

## Results and discussion

### Fabrication of NBS and selection of redox-active cathode materials

Developing an all-organic NBS with suitable catholyte and anolyte materials is challenging owing to the interplay between the solubility of the active material and its crossover. Li metal was selected as the anode material because of its high energy density and theoretical capacity (~3860 mAh/g) and low electrochemical potential (–3.04 V vs. SHE)^13^. On the other hand, graphite felt (GF) was selected as the cathode material because of its high specific surface area, porous structure, high electrical conductivity, and chemical inertness in nonaqueous electrolytes. The unique structure of GF and the presence of a large number of surface catalytic sites result in fast redox-reaction kinetics and hence improved battery performance. The successful fabrication of an NBS for high-performance membrane-free LRFBs requires that (1) the two nonaqueous electrolytes must be immiscible, show high ionic conductivities, and a wide electrochemical stability window^24,35^ and (2) the redox-active cathode material should have high solubility in the catholyte and low solubility in the anolyte in both the charging and discharging states to prevent the self-discharging of the battery because of crossover^23,36^. Both requirements are discussed below.

To explore electrolytes with the above-mentioned properties, eleven nonaqueous solvents (including four ionic and seven nonionic organic solvents) were screened to construct the NBS (Supplementary Table 1). Ionic liquids have been explored widely for use in Li-ion batteries as compatible anolytes for Li metal, as they not only allow for a wider electrochemical window (~6 V) but also suppress the growth of Li dendrites during longer battery operations^37–39^. Here, four ionic liquids, namely, ethyl-3-methylimidazolium bis(trifluoromethylsulfonyl)imide (EMI-TFSI), *N*-propyl-*N*-methylpyrrolidinium bis(trifluoromethanesulfonyl)imide (PMP-TFSI), 1-ethyl-3-methyl imidazolium tetrafluoroborate (EMI-BF_4_), and 1-butyl-1-methylpyrrolidinium bis(trifluoromethylsulfonyl)imide (BMP-TFSI), along with nonafluoro-1,1,2,2-tetrahydrohexyl-trimethoxysilane (NFTTS) were selected as the anolyte solvents, and six organic solvents, namely, tetrahydrofuran (THF), tetraethylene glycol dimethyl ether (TEGDME), fluoroethylene carbonate (FEC), diethylene carbonate (DEC), propylene carbonate (PC), and benzotrifluoride (BTF), were explored for use as the catholyte. A few pairs of the anolyte and catholyte solvents were intrinsically immiscible in their neat forms. The other electrolytes that were either miscible or partially immiscible in their neat forms could be rendered immiscible using salt-out strategies^24^. The most effective salt-out strategy involves the use of salts that have high solubility in one organic phase and low (or no) solubility in another organic phase to facilitate the formation of a biphasic system^19^. To screen the electrolyte salts with the above-mentioned properties, five salts, namely, lithium bis(trifluoromethanesulfonyl)imide (LiTFSI), lithium perchlorate (LiClO_4_), tetrabutylammonium hexafluorophosphate (TBAPF_6_), tetrabutylammonium tetrafluoroborate (TBABF_4_), and lithium hexafluorophosphate (LiPF_6_), were selected for solubility analyses. The concentration of various salts was adjusted within the range of 0.5 M to 1.5 M to achieve the separation of two organic solvents. However, the selective solubility of organic salts in different solvents resulted in only a few pairs forming a stable biphasic system, while others remained miscible or partially miscible. Following the analyses, three NBSs were formed: FEC (LiClO_4_ (1.5 M))/BMP-TFSI (LiTFSI (1 M)), TEGDME/NFTTS with LiTFSI (1 M), and BTF-TEGDME/EMI-BF_4_ with TBABF_4_ (1 M). Since both phases of the NBS should have the same exchange ions to facilitate the charging/discharging of the membrane-free battery^19,21^, Li^+^ ions were used as the exchange ions for FEC/BMP-TFSI and TEGDME/NFTTS biphasic systems, and BF_4_^-^ ions for BTF-TEGDME/EMI-BF_4_ biphasic. Of the three NBSs, FEC /BMP-TFSI (Supplementary Fig. 1) was selected for the subsequent electrochemical analyses and battery performance studies due to the following reasons. The high solubilizing ability of FEC solvent (bottom phase) allowed catholytes (Supplementary Fig. 2 and Supplementary Table 2) with different redox potentials to be evaluated. Plus, FEC exhibits higher ionic conductivity compared with those of BTF and NFTTS^40,41^. Meanwhile, BMP-TFSI solvent (top phase) facilitates improved compatibility as an anolyte electrolyte solvent for Li metal anode. In addition, the organic solvents utilized in this study as the anolyte and catholyte solvents possess high flash points of approximately 210 °C and 115 °C, respectively. For clarification, we have conducted an ignition test on both the top (BMP-TFSI/LiTFSI) and bottom (FEC/LiClO_4_) phases of the biphasic system. As illustrated in Supplementary Fig. 3, both phases were determined to have high flame resistance.

Moreover, to investigate the ion transport kinetics in different phases as well as at the liquid–liquid interface, we carried out electrochemical impedance spectroscopy (EIS) measurements for the FEC electrolyte solvent, BMP-TFSI electrolyte solvent, and the BMP-TFSI/FEC (liquid/liquid) system, which included the liquid/liquid interface. According to Jeganathan et al.^42^, the equivalent circuit of a BMP-TFSI/FEC system is composed of bulk impedance (solution and wire), FEC capacitance, BMP-TFSI capacitance (including SEI layer) and liquid–liquid interface resistance, and diffusion impedance. By separately measuring the impedance of the FEC and the BMP-TFSI electrolytes (Supplementary Fig. 4a, b), and fixing the main parameters of these two parts during the fitting process, the liquid–liquid interface impedance was calculated to be ~10.0 Ω (Supplementary Fig. 4c). The equivalent circuit fitting data for the EIS measurement are detailed in the Supplementary Table 3. It should be noted that a negative C_IF_ capacitance was obtained, as observed in other similar systems, presumably due to the delayed-current phenomenon^42,43^. However, compared to R_ct_ of over 100 Ω, an interfacial impedance of 10.0 Ω is not considered to be a limiting factor.

A catholyte with high solubility with respect to the anolyte solvent would ensure high energy density while one with low (ideally zero) solubility would prevent crossover. To explore catholyte materials that meet these solubility requirements, eleven organic compounds were selected, and their solubilities in both phases of the NBS were tested. These materials included derivatives of phenothiazine^21,35^, cyclopropenium^44,45^, 2,2,6,6-tetramethylpiperidinyloxy (TEMPO)^32,46,47^, anthraquinone (AQ)^33^, and ferrocene^48,49^ (Supplementary Fig. 2 and Supplementary Table 2). Among the catholyte materials, Tri-TEMPO, C3-PTZ, and CP showed high solubilities in cathode electrolyte (FEC/LiClO_4_) and poor affinity towards the anode electrolyte (BMP-TFSI/LiTFSI) (Supplementary Fig. 5). The solubilities of Tri-TEMPO, C3-PTZ, and CP in FEC/LiClO_4_ (1.5 M) were ~1.2 M, ~1 M, and 1.8 M, respectively. Negligible crossover occurred under static conditions over 15 days, as confirmed by ultraviolet (UV)-visible spectroscopy (Supplementary Fig. 6) and cyclic voltammetry (CV) (Supplementary Fig. 7). Hence, Tri-TEMPO, C3-PTZ, and CP were selected as the cathode materials for the subsequent experiments.

### Electrochemical characterization

**Fig. 2:**
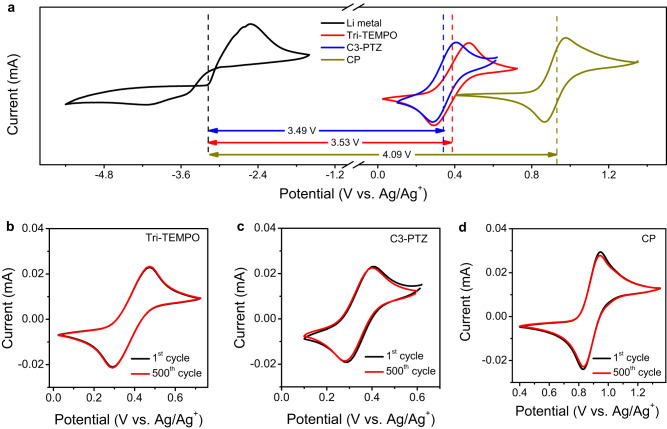
Electrochemical characterization of redox-active materials. **a** Cyclic voltammograms of Li metal in BMP-TFSI/LiTFSI (0.1 M) (with a Li-metal working electrode, a platinum counter electrode and Ag/Ag^+^ a reference electrode) and redox-active cathode materials including Tri-TEMPO (5 mM), C3-PTZ (5 mM), or CP (5 mM) in FEC/LiClO_4_ (0.1 M) solutions (with a glassy carbon (GC) working electrode, a platinum counter electrode and Ag/Ag^+^ a reference electrode). **b**–**d** Cyclic voltammetry stability of Tri-TEMPO, C3-PTZ, and CP in FEC/LiClO_4_ (0.1 M) solutions. All the CV measurements were conducted under an inert argon atmosphere (at 27 °C) at a scan rate of 50 mV/s.

The CV measurement was conducted to ascertain the electrochemical compatibility of the Li metal anode in the ionic liquid (BMP-TFSI), as well as to evaluate the reversibility of the redox-active cathode materials (Tri-TEMPO, C3-PTZ, and CP) in FEC under an inert argon atmosphere at a temperature of 27 °C. Tri-TEMPO, C3-PTZ, and CP exhibit a reversible redox couple at 0.37, 0.33, and 0.93 V vs. Ag/Ag^+^ in FEC/LiClO_4_ (0.1 M) (Fig. 2a). The pairs of the Li-metal anode and the three cathode materials (i.e., Li||Tri-TEMPO, Li||C3-PTZ, and Li||CP) exhibited theoretical cell voltages of 3.53, 3.49, and 4.09 V, respectively; these are higher than those of previously reported biphasic membrane-free batteries (Supplementary Table 4). The peak potential separations of Tri-TEMPO, C3-PTZ, and CP at a scan rate of 5 mV/s were ~74, ~69, and ~76 mV, respectively (Supplementary Fig. 8) and indicative of a one-electron redox process as per the Nernst equation^50^. Next, CV measurements were performed at scan rates of 50–200 mV/s (Supplementary Fig. 9) to study the electrochemical kinetics of the catholyte compounds. The ratios of the cathodic peak current (i_pc_) and anodic peak current (i_pa_) for Tri-TEMPO, C3-PTZ, and CP at the investigated scan rates (50–200 mV/s) were close to 1 (Supplementary Fig. 10), suggesting that the compounds exhibited good electrochemical reversibility in FEC. Further, the peak currents (anodic and cathodic) exhibit a linear relationship with the square root of the scan rate (ν^1/2^) for all three cathode materials (Supplementary Fig. 11), indicating that the redox process is diffusion-controlled^50^. In addition, linear sweep voltammetry (LSV) was also performed using the rotating disk electrode (RDE) experiments (Supplementary Figs. 12, 13, and 14) to study the electrochemical kinetics of redox molecules used in this work. The Koutecký–Lévich curves (Eq. 1) were used to measure the diffusion coefficients (*D*) of the redox materials at varying rotating rates. The diffusion coefficients of Tri-TEMPO, C3-PTZ, and CP are in the range of 10^−6^–10^−5^ cm^2^ s^−1^. Furthermore, the kinetic rate constants (*k*_*o*_) of all three redox compounds were determined by fitting the Butler–Volmer equation (Eq. 2) and were found to be in the range of 10^–4^–10^–2^ cm s^−1^. Notably, the diffusion coefficients and rate constants of these compounds are comparable to those of certain redox-active organic materials employed in aqueous flow batteries^51,52^. The fast electrode kinetics of Tri-TEMPO, C3-PTZ, and CP are anticipated to result in low activation polarization loss, which is beneficial for flow battery applications. The electrochemical and chemical stability of organic redox materials were evaluated using CV and proton nuclear magnetic resonance (^1^H NMR) spectroscopy. The CV cycling results showed improved electrochemical stability, as evidenced by the nearly superimposable voltammograms (Figs. 2b–d). In addition, the ^1^H NMR spectra of C3-PTZ and CP demonstrated improved thermo-stability over 10 days at 25 °C and 60 °C (Supplementary Figs. 15, 16).

### Charge/discharge performance of biphasic membrane-free batteries under static conditions

**Fig. 3:**
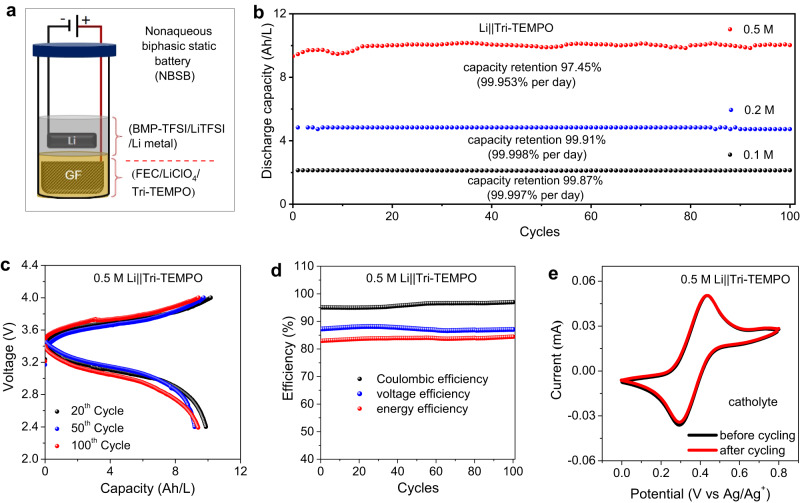
Performances of Li||Tri-TEMPO based nonaqueous biphasic static batteries (NBSBs). **a** Schematic representation of the NBSB employed in this study, depicting the placement of a Li metal anode within the anolyte solvent (BMP-TFSI/LiTFSI) and the dissolution of redox-active materials in the catholyte solvent FEC. **b** Discharge capacities of 0.1, 0.2, and 0.5 M Li||Tri-TEMPO NBSBs. **c** 20th, 50th, and 100th charge/discharge profiles of 0.5 M NBSB. **d** Coulombic efficiency, voltage efficiency, and energy efficiency of 0.5 M NBSB. **e** Cyclic voltammograms of 0.5 M NBSB before and after 100 charge/discharge cycles (catholyte). The measurements were conducted under an inert argon atmosphere (at 27 °C), with long-term cycling of batteries performed at a current density of 1 mA/cm². CV measurements were carried out at a scan rate of 50 mV/s.

The viability of the developed biphasic system as a membrane-free battery was initially evaluated under static conditions under an inert argon atmosphere (27 °C). Supplementary Fig. 17 presents a digital image of the static cell, providing a visual depiction of its detailed components. The corresponding schematic illustration, Fig. 3a, offers a clear representation of the cell’s structural arrangement and functional elements. As a starting point, two nonaqueous biphasic static batteries (NBSBs) were assembled by pairing a Li-metal anode with 0.1 or 0.2 M Tri-TEMPO, and the batteries were subjected to charging/discharging for 100 cycles (~13 days for 0.1 M Tri-TEMPO and ~22 days for 0.2 M Tri-TEMPO) at a current density of 1 mA/cm^2^. While the current density could be increased further, the operational current density was notably higher than what has been previously reported for aqueous and nonaqueous biphasic membrane-free systems^23,24^. The 0.1 M and 0.2 M Li||Tri-TEMPO NBSBs (Fig. 3b) both showed good cycling performances, exhibiting capacity retention rates of 99.87% (99.998% per cycle, 99.997% per day) and 99.91% (99.998% per cycle, 99.998% per day), respectively. This confirmed that the redox-active materials, supporting electrolytes, and solvent exhibited high stability under actual battery operation conditions. Moreover, both NBSBs exhibited high CE (over 99% for both) and EE values (86% for 0.1 M and 84% for 0.2 M). A CE value as high as ~99% suggests low self-discharging and thus negligible active material crossover. Moreover, there was no obvious change in the peak current densities of the 0.1 M and 0.2 M Li||Tri-TEMPO catholytes (Supplementary Fig. 18) post-cycling, which further confirmed that the Tri-TEMPO redox molecules remained confined within the catholyte (FEC phase) during the prolonged cycling process. Based on these results, we built an NBSB containing the catholyte in a higher concentration. Specifically, a 0.5 M Li||Tri‒TEMPO NBSB was assembled and subjected to long-term charging/discharging (100 cycles, ~55 days) at a current density of 1 mA/cm^2^ (27 °C), as shown in Fig. 3b. The 0.5 M Li||Tri‒TEMPO battery exhibited a capacity retention rate of 97.5% (99.97% per cycle, 99.95% per day). Its cycling profile suggests a discharging voltage of 3.48 V with no significant change in the overpotential throughout the cycling process (Fig. 3c). The 0.5 M Li||Tri-TEMPO NBSB displayed CE, VE, and EE values of 96%, 87%, and 83% (Fig. 3d), respectively, which surpass those of previously reported membrane-free systems (Supplementary Table 4). However, these values were lower than those of the 0.1 M and 0.2 M NBSBs (Supplementary Fig. 19). The Coulombic efficiency of a high-concentration battery did not change with higher current density. Even though faster charging/discharging rates typically increase Coulombic efficiency, parasitic reactions could also occur at higher current density as a tradeoff. Moreover, a higher catholyte concentration would mean a relatively high flow resistance, resulting in lower ionic conductivity and greater transport losses, and consequently a lower VE value^53^. However, the 0.5 M Tri-TEMPO NBSB had a discharge volumetric energy density of 34 Wh/L which is higher than those of most state-of-the-art biphasic membrane-free static and flow batteries (Supplementary Table 4). The electrolytes were analyzed both before and after cycling using CV (Fig. 3e, Supplementary Fig. 20a) and UV-visible spectroscopy (Supplementary Fig. 20b). The analyses indicated that Tri-TEMPO was not present in the post-cycling anolyte. Hence, the high cycling stability of the Li||Tri-TEMPO biphasic static batteries with different Tri-TEMPO concentrations (0.1, 0.2, and 0.5 M) suggested that they are suited for practical use. It also confirmed the high compatibility of Li metal with the anolyte and that of GF with the catholyte.

**Fig. 4:**
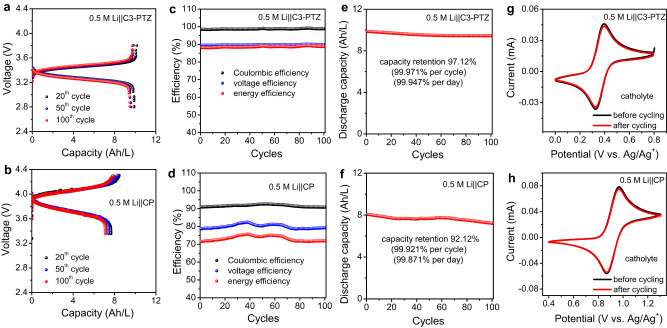
Charge/discharge performances of 0.5 M Li||C3-PTZ and 0.5 M Li||CP-based nonaqueous biphasic static batteries (NBSBs). **a**, **b** Charge/discharge profiles of 0.5 M C3-PTZ and 0.5 M CP NBSBs after 20th, 50th, and 100th cycles. **c**, **d** Coulombic efficiency, voltage efficiency, and energy efficiency values of 0.5 M C3-PTZ and 0.5 M CP NBSBs. **e**, **f** Discharge capacities of 0.5 M C3-PTZ and 0.5 M CP NBSBs. **g**, **h** Cyclic voltammograms of 0.5 M C3-PTZ and 0.5 M CP NBSBs before and after 100 charge/discharge cycles (catholyte). The measurements were conducted under an inert argon atmosphere (at 27 °C), with long-term cycling of batteries performed at a current density of 1 mA/cm². CV measurements were carried out at a scan rate of 50 mV/s.

To evaluate the versatility of the developed NBS, the other two selected catholyte materials (C3-PTZ and CP) were studied under the same conditions as those used for the Li||Tri-TEMPO battery (Fig. 4). The long-term performances of both NBSBs were investigated through extended charging/discharging tests performed at 1 mA/cm^2^ for 100 cycles. The 0.5 M Li||C3-PTZ battery was charged/discharged within the voltage range of 2.8–3.8 V (Fig. 4a), whereas the 0.5 M Li||CP battery was cycled in the 3.3–4.3 V range (Fig. 4b). The 0.5 M Li||C3-PTZ and 0.5 M Li||CP NBSBs exhibited distinct stable plateaus and discharging voltages of 3.42 and 3.94 V, respectively, which are considerably greater than those reported in the majority of membrane-free flow battery systems, whether aqueous or nonaqueous (Supplementary Table 4). The 0.5 M Li||C3-PTZ (Fig. 4c) battery also showed stable CE, VE, and EE values of 98%, 90%, and 88%, respectively, throughout the cycling process, whereas the 0.5 M Li||CP (Fig. 4d) battery exhibited CE, VE, and EE values of 91%, 79%, and 72%, respectively. That the CE values of both batteries were greater than 90% indicated that negligible self-discharging had occurred in the batteries Furthermore, the 0.5 M Li||C3-PTZ (Fig. 4e) and 0.5 M Li||CP (Fig. 4f) batteries showed good cycling stability, with their capacity retention rates being 97.12% (99.971% per cycle, 99.949 per day) and 92.12% (99.921 per cycle, 99.856 per day), respectively. The electrolytes of the 0.5 M Li||C3-PTZ and 0.5 M Li||CP batteries were subjected to CV measurements, UV-visible spectroscopy, and proton nuclear magnetic resonance (^1^H NMR) spectroscopy both before and after cycling. The quantitative CV analysis of the catholytes and anolytes of the 0.5 M Li||C3-PTZ (Fig. 4g, Supplementary Fig. 21a) and 0.5 M Li||CP (Fig. 4h, Supplementary Fig. 22a) batteries did not indicate significant changes in their current densities even after 100 charge/discharge cycles. Moreover, the post-cycling UV-visible spectra of the 0.5 M Li||C3-PTZ and 0.5 M Li||CP anolytes confirmed the absence of crossover (Supplementary Figs. 21b, 22b). Finally, the NMR spectra of the pre-and post-cycled anolytes of both batteries (Supplementary Figs. 21c, 22c) were nearly identical, which confirmed that C3-PTZ and CP remained in the catholyte phases and did not cross over.

**Fig. 5:**
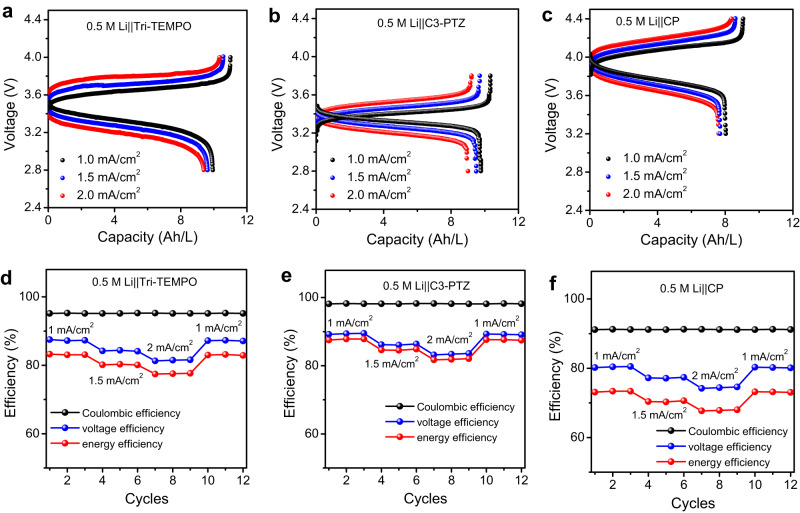
Performance of Tri-TEMPO, C3-PTZ, and CP-based nonaqueous biphasic static batteries (NBSBs) at different current densities. **a**–**c** Charge/discharge profiles of 0.5 M Li||Tri-TEMPO, 0.5 M Li||C3-PTZ, and 0.5 M Li||CP NBSBs at different operating current densities. **d**–**f** Variation in Coulombic efficiency, voltage efficiency, and energy efficiency of 0.5 M Li||Tri-TEMPO, 0.5 M Li||C3-PTZ, and 0.5 M Li||CP NBSBs at different operating currents densities. The measurements were conducted under an inert argon atmosphere (at 27 °C).

To elucidate the effect of the high current density of the NBSBs on their performance, the charge/discharge behaviors of the 0.5 M Li||Tri-TEMPO, 0.5 M Li||C3-PTZ, and 0.5 M Li||CP batteries were studied at current densities of 1, 1.5, and 2 mA/cm^2^, and their average CE, VE, and EE values were measured for 3 cycles. The 0.5 M Li||Tri-TEMPO (Fig. 5a), 0.5 M Li||C3-PTZ (Fig. 5b), and 0.5 M Li||CP (Fig. 5c) NBSBs exhibited ohmic drops of ~80, ~50, and ~60 mV, respectively, at 1.5 mA/cm^2^ and ~130, ~98, and ~110 mV, respectively, at 2 mA/cm^2^. The increase in the ohmic loss at the higher current density can be ascribed to limitations related to mass diffusion^54^. The Li||Tri-TEMPO (Fig. 5d), Li||C3-PTZ (Fig. 5e), and Li||CP (Fig. 5f) batteries showed CE values of 96%, 98%, and 92%, respectively, at all the operating current densities, indicating that fast charging/discharging did not induce noticeable side reactions. However, the EE values of the Li||Tri-TEMPO, Li||C3-PTZ, and Li||CP batteries were reduced from 83%, 88%, and 72%, respectively, to 78%, 80%, and 70%, when the applied current density was increased from 1 to 2 mA/cm^2^. This was owing to an increase in the overpotential because of mass transport losses^54^. It should be noted that all three batteries regained their original efficiencies when they were cycled back at 1 mA/cm^2^, thus confirming their improved charge-rate performance. In addition to this, the polarization for the 0.5 M Li||Tri-TEMPO, C3-PTZ, and CP batteries under static conditions was also investigated at different states of charge (SOC). The peak power densities of the 0.5 M Li||Tri-TEMPO, C3-PTZ, and CP batteries under static conditions are 33, 30, and 37 mW/cm^2^, respectively, at 100% SOC (Supplementary Fig. 23). Correspondingly, the area-specific resistance (ASR) data for both the resistance of the electrolyte and the whole cell under static conditions was also studied. The results show that both the ASR of the electrolyte and the whole cell increase as the operating current density increases (Supplementary Fig. 24). However, the electrolyte resistance accounts for over 50% of the whole cell for all batteries. In addition, the energy densities of the static 0.5 M Li||Tri-TEMPO, C3-PTZ, and CP batteries were 34, 34, and 30 Wh/L, respectively. These values were found to be 71%, 72%, and 57% of their corresponding theoretical energy densities (Supplementary Table 4).

### Charge/discharge performance of biphasic membrane-free batteries under flow conditions

**Fig. 6:**
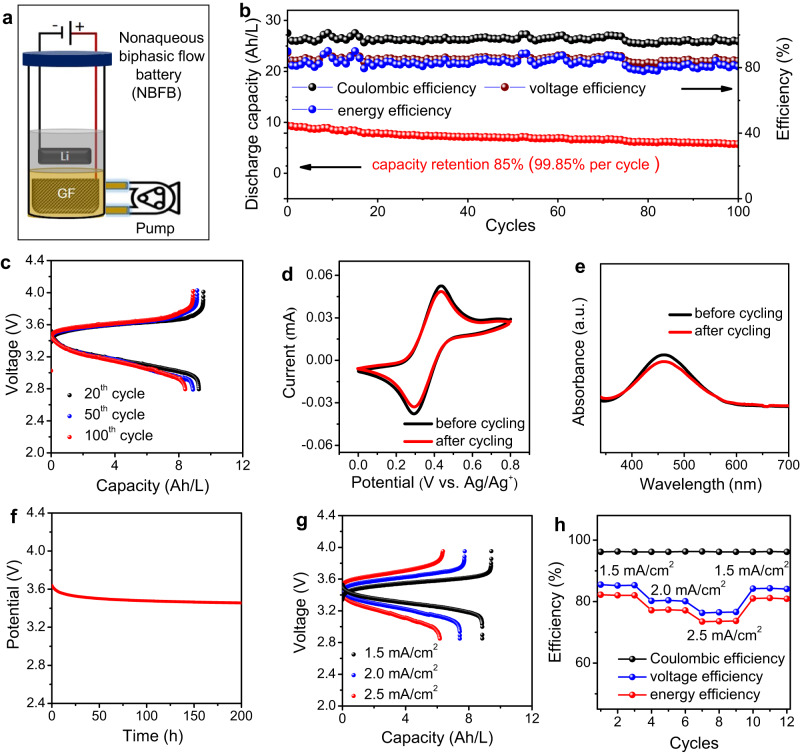
Performances of 0.5 M Li||Tri-TEMPO based membrane-free nonaqueous biphasic flow batteries (NBFBs). **a** Schematic of membrane-free NBFB used in this study. **b** Capacity retention rate, Coulombic efficiency, voltage efficiency, and energy efficiency of NBFB. **c** Charge/discharge profile after 20th, 50th, and 100th cycles of NBFB. **d** Cyclic voltammograms of catholyte of NBFB before and after 100 charge/discharge cycles. **e** UV-visible spectra of catholyte of NBFB before and after 100 charge/discharge cycles. **f** Voltage change in of fully charged NBFB over time. **g** Charge/discharge profiles at different current densities. **h** Charge-rate performance of NBFB. The measurements were conducted under an inert argon atmosphere (at 27 °C), with long-term cycling of batteries performed at a current density of 1.5 mA/cm². CV measurements were carried out at a scan rate of 50 mV/s.

While the batteries showcased impressive performance under static conditions, their energy and power remained intertwined, limiting their full potential. Thus, the capacity of the batteries was not scalable, as is necessary under flow conditions. Hence, a Li-based nonaqueous biphasic flow battery based on 0.5 M Tri-TEMPO was assembled. Supplementary Fig. 25 presents a comprehensive digital photograph, while Fig. 6a provides a schematic illustration, both showcasing the components of a membrane-free biphasic flow battery. The long-term cycling performance was evaluated under an inert argon atmosphere (at 27 °C) at the current density of 1.5 mA/cm^2^. Under flow conditions (1 mL/min), after 100 charge/discharge cycles, the 0.5 M Li||Tri-TEMPO battery showed a capacity retention rate of 81.12% (99.811 per cycle, 99.581 per day) along with a CE of ~96%, VE of ~85%, and EE of ~82% (Fig. 6b, c). To elucidate the mechanism responsible for its capacity fading, its electrolytes were analyzed both before and after cycling using CV (Fig. 6d) and UV-visible spectroscopy (Fig. 6e). The reduction in the CV peak current and absorbance of the post-cycling catholyte were indicative of the crossover of the Tri-TEMPO molecules due to the convective diffusion of the flowing catholyte during prolonged battery cycling. Both CV and UV-visible results (Supplementary Fig. 26) suggested that 93% of total capacity fade is caused by the crossover of Tri-TEMPO. To clarify the effect of the flowing catholyte on the self-discharging of the battery, its open-circuit voltage (OCV) in the fully charged state was monitored over 200 h (at 27 °C) (Fig. 6f). The OCV of the battery displayed a sharp decrease during the first 10 h after the charging process owing to ohmic polarization (i_R_ drop), followed by a voltage loss of 0.63 mV/h in the next 190 h, which corresponded to a total drop of 120 mV over 190 h. However, the per-hour voltage loss of this battery was smaller than that of previously reported aqueous/nonaqueous biphasic membrane-free batteries^19,22^. Further, the ASR result for both the resistance of the electrolyte and the whole cell under flow conditions suggests that the electrolyte resistance accounts for over 50% of the whole battery (Supplementary Fig. 24). Nonetheless, for the 0.5 M Li||Tri-TEMPO battery, both the electrolyte and overall battery’s ASR exhibit a notable decrease under flow conditions. This reduction in ASR under flow conditions enables the battery to achieve higher current and power densities without compromising its performance. Hence, the peak power density of the 0.5 M Li||Tri-TEMPO battery under flow conditions was significantly higher at 61 mW/cm^2^ compared to the Tri-TEMPO static battery (Supplementary Fig. 23). The enhanced peak power density can be attributed to improved mass transport under flow conditions.

**Fig. 7:**
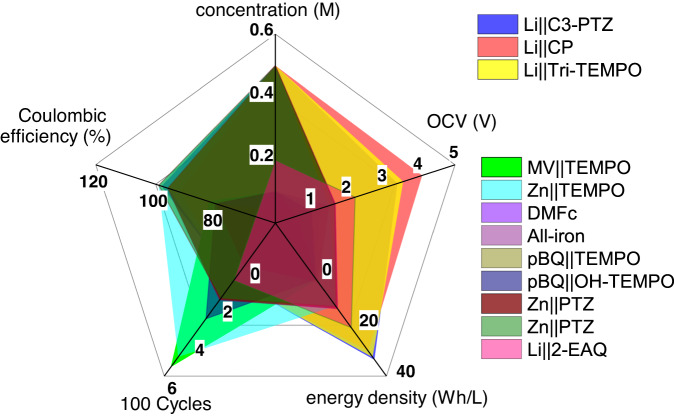
Comprehensive comparison of different battery performances. Radar chart comparing the performance of our demonstrated batteries with the performance of other biphasic battery systems: MV||TEMPO^24^, Zn||TEMPO^19^, DMFc^27^, All-iron^66^, pBQ||TEMPO^67^, pBQ||OH-TEMPO^67^, Zn/PTZ^21^, Zn||PTZ^22^, Li||2-EAQ^23^.

The charge-rate performance of the 0.5 M Li||Tri-TEMPO flow battery was evaluated at current densities of 1.5, 2.0, and 2.5 mA/cm^2^, and the corresponding cycling profiles (Fig. 6g) as well as the CE, VE, and EE (Fig. 6h) values were measured for an average of three cycles. When operated at a current density of 2.5 mA/cm^2^, the battery exhibited a discharge overpotential of 132 mV, which is nearly twice that when it was operated at a current density of 1.5 mA/cm^2^. The increase in the overpotential and the corresponding reduction in the VE at the higher current density can be attributed to mass transport limitations^54^. However, the battery was able to regain its original performance when cycled again at the applied current density of 1.5 mA/cm^2^, suggesting that the biphasic battery showed good rate cyclability under flow conditions. Hence, the performance of membrane-free nonaqueous biphasic batteries demonstrated in this study, under both static and flow conditions, is well positioned compared to the state-of-the-art literature of similar battery systems (Supplementary Table 4). A radar chart in Fig. 7 summarizes this work and reported battery performance, including number of cycles, energy density, Coulombic efficiency, and voltage.

To evaluate the economic feasibility of the developed system, Supplementary Note 1 and Supplementary Table 5 present a cost estimation of the battery components and chemicals employed in this study. This economic analysis provides comprehensive information regarding the cost analysis of the redox-active materials utilized in this research. The findings indicate that the synthesized redox-active cathode materials can be prepared on a larger scale at economically favorable prices, further supporting their viability.

In summary, we report a nonaqueous biphasic membrane-free Li-based redox flow battery with high voltage and energy density. A nonaqueous biphasic system was developed using an ionic liquid (BMP-TFSI) and organic carbonate as the electrolytes (FEC) based on the salt-out effect. Three redox-active cathode materials, namely, Tri-TEMPO, C3-PTZ, and CP, were selected after analyzing 11 organic redox-active compounds. Initially, the 0.1 M and 0.2 M Li||Tri-TEMPO static biphasic membrane-free batteries were subjected to 100 charge/discharge cycles and showed capacity retention rates of 99.87% and 99.91%, respectively. The 0.1 M and 0.2 M Li||Tri-TEMPO biphasic batteries exhibit energy densities of ~7.2 and 16.6 Wh/L, respectively. To increase the energy density, a 0.5 M Li||Tri-TEMPO membrane-free static battery was assembled and subjected to 100 cycles. It exhibited a capacity retention rate of ~98%, CE of 96%, and energy density of 34 Wh/L. To confirm the versatility of the proposed biphasic system, two more redox-active materials, namely, C3-PTZ and CP, were tested in a membrane-free battery under static conditions. The 0.5 M Li||C3-PTZ and 0.5 M Li||CP biphasic static batteries exhibited discharge voltages of 3.42 and 3.94 V, respectively, which were higher than those of previously reported biphasic membrane-free battery systems. The Li||C3-PTZ and Li||CP biphasic static batteries containing the redox-active materials in a 0.5 M concentration displayed capacity retention rates of 98%, and 92%, respectively, after 100 cycles over ~55 days. Post-cycling analyses indicated that there were no signs of material degradation or crossover even after prolonged battery operation for any of the membrane-free static batteries. This confirmed the versatility of the nonaqueous biphasic system. To ensure the decoupling of energy and power, the developed membrane-free batteries were also studied under flow conditions. The representative 0.5 M Li||Tri-TEMPO flow battery exhibited a capacity retention rate of 81% after 100 cycles over ~45 days, with its CE and EE values being 96% and 82%, respectively. The flow biphasic battery displayed higher energy density (33 Wh/L) than those of the earlier reported membrane-free batteries. The peak power densities of the 0.5 M Li||Tri-TEMPO, C3-PTZ, and CP batteries under static conditions are 33, 30, and 37 mW/cm^2^, respectively, at 100% SOC. The peak power density of the 0.5 M Li||Tri-TEMPO battery under flow conditions was higher at 61 mW/cm^2^ compared to the Tri-TEMPO static battery. In future work, several strategies can be implemented to improve the system’s kinetics and overall performance, including: (1) optimizing the flow rates within the battery to enhance mass transfer kinetics; (2) selecting suitable supporting electrolyte (salt) that can improve the electrolyte’s conductivity to regulate the overall battery kinetics, (3) implementing an advanced cell design for both static and flow conditions to reduce the dead volume of the cathode electrode, to improve capacity utilization, and (4) mitigating crossover of cathode materials from catholyte to anolyte at the liquid/liquid interface under flow conditions to further improve Coulombic efficiency.

## Methods

### Materials

Lithium hexafluorophosphate (LiPF_6_, 99.99%) was purchased from Sigma Aldrich. The ILs used, namely, 1-Ethyl-3-methylimidazolium Bis(trifluoromethanesulfonyl)imide (EMI-TFSI, 98.0%), *N*-propyl-*N*-methylpyrrolidinium bis(trifluoromethanesulfonyl)imide (PMP-TFSI, 99.9%), 1-ethyl-3-methyl imidazolium tetrafluoroborate (EMI-BF_4_, 97.0%), and 1-butyl-1-methylpyrrolidinium bis(trifluoromethylsulfonyl)imide (BMP-TFSI, 98%), were purchased from TCI Chemicals, all the ionic liquids were used as received (water impurity content was not measured). Lithium perchlorate (LiClO_4_, 98%), lithium bis(trifluoromethanesulfonyl)imide (LiTFSI, 99.94%), fluoroethylene carbonate (FEC, 99%), diethyl carbonate (DEC, 99%), propylene carbonate (PC, 99.7%), nonafluoro-1,1,2,2-tetrahydrohexyl-trimethoxysilane (NFTTS, 97%), benzotrifluoride (BTF, 99.8%), Tetrahydrofuran (THF, 99.9), and tetraethylene glycol dimethyl ether (TEGDME, 98.0%) were purchased from Fisher Scientific and used without further purification. Tetrabutylammonium hexafluorophosphate (TBAPF_6_, 99.0%) was recrystallized in anhydrous ethanol and dried under vacuum at 60 °C for 24 h. The other compounds used in this study, such as Tri-TEMPO^55^, C3-PTZ^56^, CP^57,58^, C8-PTZ^59^, C18-PTZ^60^, PEG3-PTZ^61^, PEG12-PTZ^56^, PEG3-TTF^62,63^, and PEG12-AQ^64^ were prepared as per the reported procedures. To prepare nonaqueous electrolyte solutions for the cathode or anode, the following procedure was followed^65^. Initially, a glass vial was filled with 3 mL of the desired electrolyte solution, either FEC or BMP-TFSI. Next, the appropriate amount of electrolyte salt was carefully added to the electrolyte solution in the glass vial. For FEC, LiClO_4_ (1.5) was added, while for BMP-TFSI, LiTFSI (1 M) was used. The entire process took place inside an argon-filled glove box, maintaining a temperature of 27 °C. To achieve a homogeneous solution, the contents of the vial were gently shaken and then subjected to ultra-sonication for 10 min. This combination of moderate shaking and ultra-sonication resulted in a uniformly mixed, clear solution, which was subsequently utilized for further analysis.

### Physicochemical characterizations

The UV-visible absorption spectra were recorded using a UV-visible spectrophotometer (Cary 8454 UV-Vis, Agilent Technologies). To perform UV-vis measurements, samples with concentrations below 5 mM were prepared. To achieve this, highly concentrated samples were diluted in an appropriate electrolyte solvent before conducting the UV-vis measurements. For the UV-vis calibration curve, stock solutions of Tri-TEMPO at concentrations of 0.3 M and 0.5 M were prepared in FEC/LiClO_4_ (1.5 M). These solutions were then further diluted to prepare solutions with concentrations of 1, 2, 3, 4, and 5 mM. In addition, a blank sample containing no redox materials was prepared to measure the UV-vis spectrum. The absorption intensity of the UV-vis spectra at different concentrations was used to construct the UV-vis calibration curve. This curve was subsequently utilized to determine the unknown concentration of the redox-active materials due to crossover.

The ^1^H NMR spectra were recorded on a Bruker AV400 spectrometer (400 MHz). The chemical shifts were reported in ppm. To determine if CP or C3PTZ had crossed through the biphasic layer, we extracted 50 mL samples from anolyte before and after subjecting the system to extended charge/discharge cycles (~100 cycles). These 50 mL samples were subjected to ^1^H NMR analysis by combining them with 250 mL of CD_3_CN, resulting in a total NMR solvent volume of 300 mL. In addition, we followed specific sample preparation procedures to assess the stability of CP and C3-PTZ using ^1^H NMR. Approximately 5 mg of either solid CP or C3-PTZ was mixed with 400 mL of CD_3_CN to create NMR samples. Two NMR samples were prepared for each compound in NMR tubes equipped with J. Young air-inlet valves. Firstly, we recorded ^1^H NMR spectra for these NMR samples, after which we stored one NMR tube of each sample at room temperature while the other was placed at 60 °C. Subsequently, we collected a series of ^1^H NMR spectra at 24-h intervals for 10 days using these samples.

### Electrochemical characterizations

For the CV measurements, 5 mM of Tri-TEMPO, C3-PTZ, and CP solutions were prepared in FEC/LiClO_4_ (0.1 M) electrolyte. A glassy carbon disk (CHI Instrument, 3 mm) was used as the working electrode and was polished with Al_2_O_3_ (Pine Research Instrumentation, Inc., 5 mm) before the measurements, where water was used as the solvent to polish the electrode. A piece of Pt wire (Pine Research Instrumentation, Inc., thickness 0.5 mm, purity 99.99%) was used as the counter electrode while an Ag/Ag^+^ (Fisher Scientific) electrode was used as the reference electrode. The CV measurements were performed using a Bio-Logic potentiostat. Electrochemical impedance spectroscopy (EIS) was performed using a potentiostatic signal spanning from 200 KHz to 100 mHz, with an amplitude of 10 mV. The measurements were conducted under open-circuit voltage (OCV) conditions for a total of 54 data points. The charge/discharge performances were evaluated using Land and Bio-Logic potentiostats.

The area-specific resistance (ASR) of both static and flow cells was determined using the linear sweep voltammetry (LSV) technique. LSV experiments were conducted on batteries containing different redox materials, and the resulting polarization curves were generated by plotting the current (i) against the applied potential (V). By identifying the linear region of the polarization curve, where the current response is directly proportional to the applied potential, the differential resistance (dE/di) was obtained. This linear region is typically observed at low overpotentials, indicating a more kinetic-limited regime of electrochemical reactions.

To calculate ASR, the slope (S) of the linear region was determined. The ASR was then computed using the formula (ASR = 1/S). ASR represents the resistance normalized to the electrode area.

Two separate sets of cells were examined for the same experiment to assess and emphasize the reproducibility of the experiment. All electrochemical characterizations were conducted at a temperature of 27 °C within an argon-filled glove box.

Linear sweep voltammetry (LSV) studies were carried out using a Pine modulated speed rotator with Biologic potentiostats. Rotating disk electrode (RDE, diameter: 5 mm), Pt wire electrode, and Ag/Ag^+^ electrode were used as the working, counter, and reference electrodes, respectively. Before testing, the samples were purged with argon (purity: 99.999%) for 10 min. LSV dates were collected at different rotation rates ranging from 100 to 1600 rpm. The diffusion coefficient (*D*_*o*_) of electroactive materials was calculated from the Lévich plot using Eq. 1^50^;

1 $$i=0.62{{{{{\rm{nFA}}}}}}{{{{{{\rm{C}}}}}}}_{{{{{{\rm{o}}}}}}}{{{{{{\rm{D}}}}}}}^{2/3}{{{{{{\rm{\omega }}}}}}}^{1/2}{{{{{{\rm{\upsilon }}}}}}}^{-1/6}$$

Where *i* is limiting current density, n is the number of electrons in the redox process, F is Faraday’s constant, A is the area of the glassy carbon electrode, C_0_ is the concentration of active material, *ω* is angular rotation rate, and *υ* is the kinematic viscosity of FEC/LiClO_4_ (0.1 M).

The kinetic rate constant is calculated by Eq. 2.

2 $${{{\mbox{i}}}}_{0}={{\mbox{FA}}}{{{\mbox{C}}}}_{0}{{{\mbox{k}}}}_{0}$$

Where i_0_ was calculated from the fitting line of the Butler–Volmer equation, the x-intercept is the log of the exchange current i_0_ (0.0003 A), F is Faraday’s constant, A is the area of the glassy carbon electrode (0.196 cm^2^), C_0_ is the concentration of redoxmers (0.5 × 10^−6^ mol/cm^3^), k_0_ is reaction rate constant (cm/s).

A biphasic system comprising FEC/LiClO_4_ (1.5 M) as the catholyte and BMP-TFSI/ LiTFSI (1.0 M) as the anolyte was utilized to assemble membrane-free static and flow batteries. To prepare the electrolyte solution, LiClO_4_ (1.5 M) salt was added to FEC and LiTFSI (1 M) salt was added to BMP-TFSI, each in separate glass vials within an argon-filled glove box. Homogeneous mixing was achieved through physical stirring, resulting in clear solutions of FEC/LiClO_4_ (1.5 M) and BMP-TFSI/LiTFSI (1.0 M). These prepared electrolyte solvents were then used to construct the biphasic battery system. The charge/discharge performances of the Li||Tri-TEMPO (0.1 M, 0.2 M, and 0.5 M), Li||C3-PTZ (0.5 M), and Li||CP (0.5 M) batteries were evaluated in the voltage ranges of 2.6–4.0, 2.8–3.8, and 3.3–4.3 V, respectively. The membrane-free batteries were tested in a cylindrical tube. For the static analyses, 0.25 mL of the catholyte and 0.25 mL of the anolyte were used. For the flow analyses, 0.6 mL of the catholyte and 0.6 mL of the anolyte were used; the catholyte was circulated through the cell at a flow rate of 1 mL/min using a peristaltic pump. The rate performances of the static and flow batteries were tested at current densities of 1, 1.5, and 2 mA/cm^2^ and 1.5, 2.0, and 2.5 mA/cm^2^, respectively. Three charge/discharge cycles were performed for each current density. The self-discharging performance of the 0.5 M Li||Tri-TEMPO membrane-free flow battery was tested by recording the OCV over time using a fully charged battery. The electrochemical measurements were conducted within an argon-filled glove box, ensuring a controlled environment with minimal oxidative reactions. The water content in the glove box was maintained below 1 ppm, while the oxygen level was kept below 2 ppm.

### Measurement of capacity utilization and retention

The capacity utilization was determined by the ratio of the operational battery capacity (C_b_) to the theoretical capacity (C_t_) at a particular state of charge (SOC). The SOC was defined based on the duration of battery charge or discharge cycles.

$${{{{{\rm{Capacity}}}}}}\;{{{{{\rm{ utilization}}}}}}\,(\%)=[{{{{{{\rm{C}}}}}}}_{{{{{{\rm{b}}}}}}}({{{{{\rm{charge}}}}}}\; {{{{{\rm{or}}}}}}\; {{{{{\rm{discharge}}}}}})/{{{{{{\rm{C}}}}}}}_{{{{{{\rm{t}}}}}}}]\times 100$$

Capacity retention was determined by normalizing the discharge capacity during cycling, where C_i_ denotes the discharge capacity obtained in the first cycle and C_f_ represents the discharge capacity obtained in subsequent cycles.

$${{{{{\rm{Capacity}}}}}}\;{{{{{\rm{retention}}}}}}\,(\%)=[{{{{{{\rm{C}}}}}}}_{{{{{{\rm{f}}}}}}}/{{{{{{\rm{C}}}}}}}_{{{{{{\rm{i}}}}}}}]\times 100$$

### Solubility test

A solution of FEC/LiClO_4_ (1.5 M) was used for the solubility measurements under an inert argon atmosphere at a temperature of 27 °C. The solution was gradually added to a vial containing known amounts of the organic compounds used as the catholytes (Tri-TEMPO, C3-PTZ, and CP). Once a clear solution was obtained, the volume (50 µL) of the electrolyte was determined using an analytical pipette (P20). The solubilities of the organic compounds were calculated from the ratio of the molar amount to the electrolyte volume. These processes were repeated twice, and the average values are presented.

### Reporting summary

Further information on research design is available in the Nature Portfolio Reporting Summary linked to this article.

### Supplementary information

Supplementary Information

Reporting Summary

## Data Availability

Most data supporting the findings of this study are included in the main text of the article and its Supplementary Information. Raw datasets can be obtained from the corresponding author on request.
