# Supplementary material for: Development of high-voltage and high-energy membrane-free nonaqueous lithium-based organic redox flow batteries
Source: Nat Commun. 2023 Aug 8;14:4753. doi: 10.1038/s41467-023-40374-y (PMC10409715; doi:10.1038/s41467-023-40374-y)
Supplement: Supplementary file 1 — Supplementary Information [file 41467_2023_40374_MOESM1_ESM.pdf]

Supplementary Information

Development of high-voltage and high-energy membrane-free nonaqueous lithium-based organic redox flow batteries

Rajeev K. Gautam, Xiao Wang, Amir Lashgari, Soumalya Sinha, Jack McGrath, Rabin Siwakoti, Jianbing "Jimmy" Jiang*

Department of Chemistry, University of Cincinnati, P.O. Box 210172, Cincinnati, Ohio 45221, United States

*Corresponding author: Email: jianbing.jiang@uc.edu

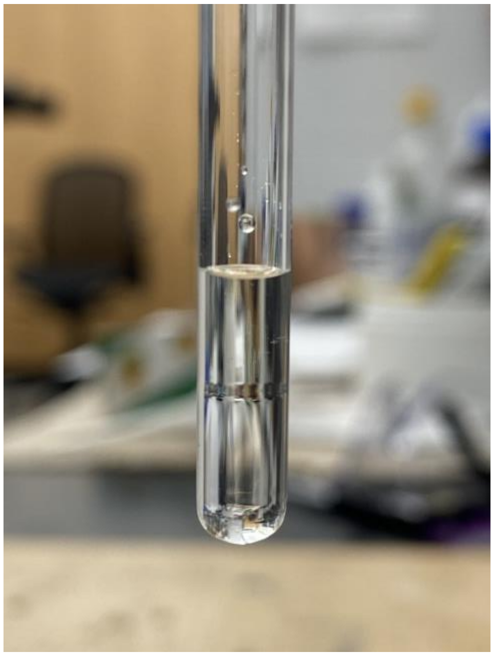

Supplementary Figure 1. Digital image showing the nonaqueous biphasic system (NBS) consisting of FEC/BMP-TFSI. The upper phase of the system contains BMP-TFSI ionic liquid (anode electrolyte), while the lower phase contains FEC solvent (cathode electrolyte). The tube containing the electrolytes is confined in a gas-inert environment.

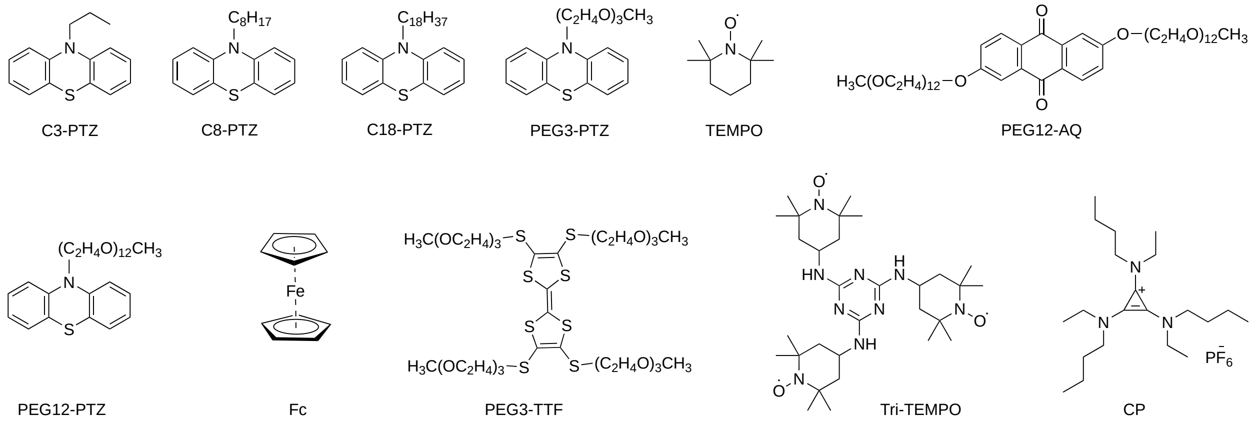

Supplementary Figure 2. The chemical structure of redox-active cathode materials was investigated for a crossover study using the newly developed biphasic system, aiming to identify an appropriate redox couple.

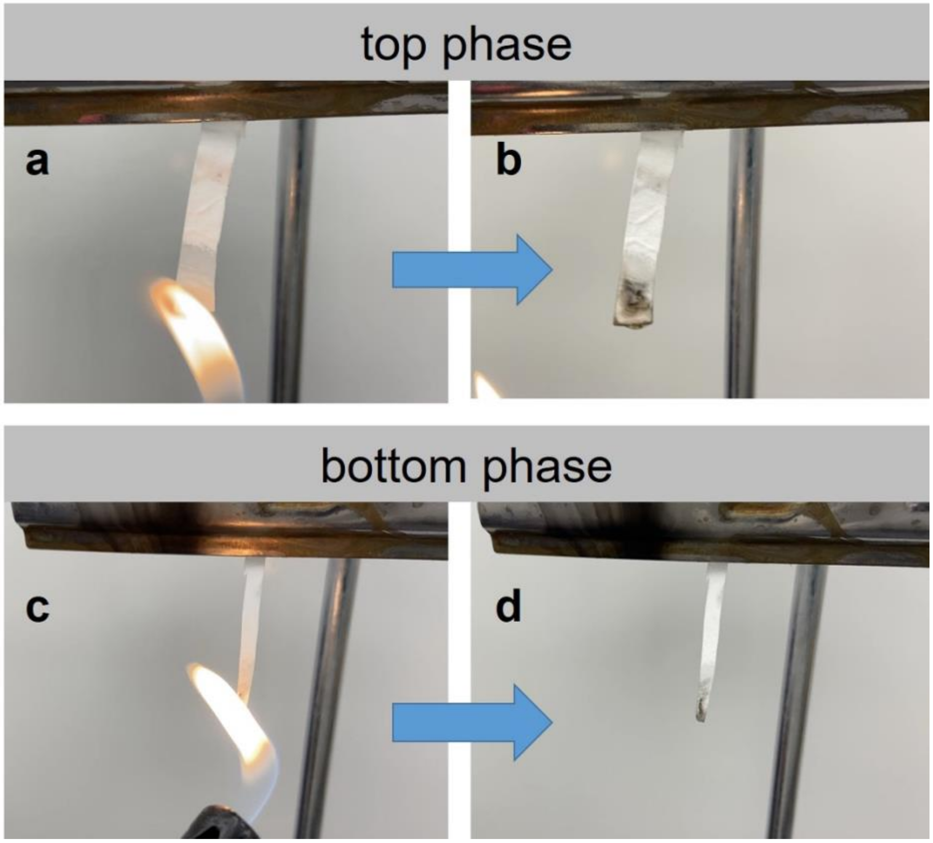

Supplementary Figure 3. Digital images showing ignition tests of glass fibers with electrolytes conducted in an ambient air atmosphere. Top phase (BMP-TFSI/LiTFSI) (a) before and (b) after the test. Bottom phase (FEC/$LiClO_4$) (c) before and (d) after the test.

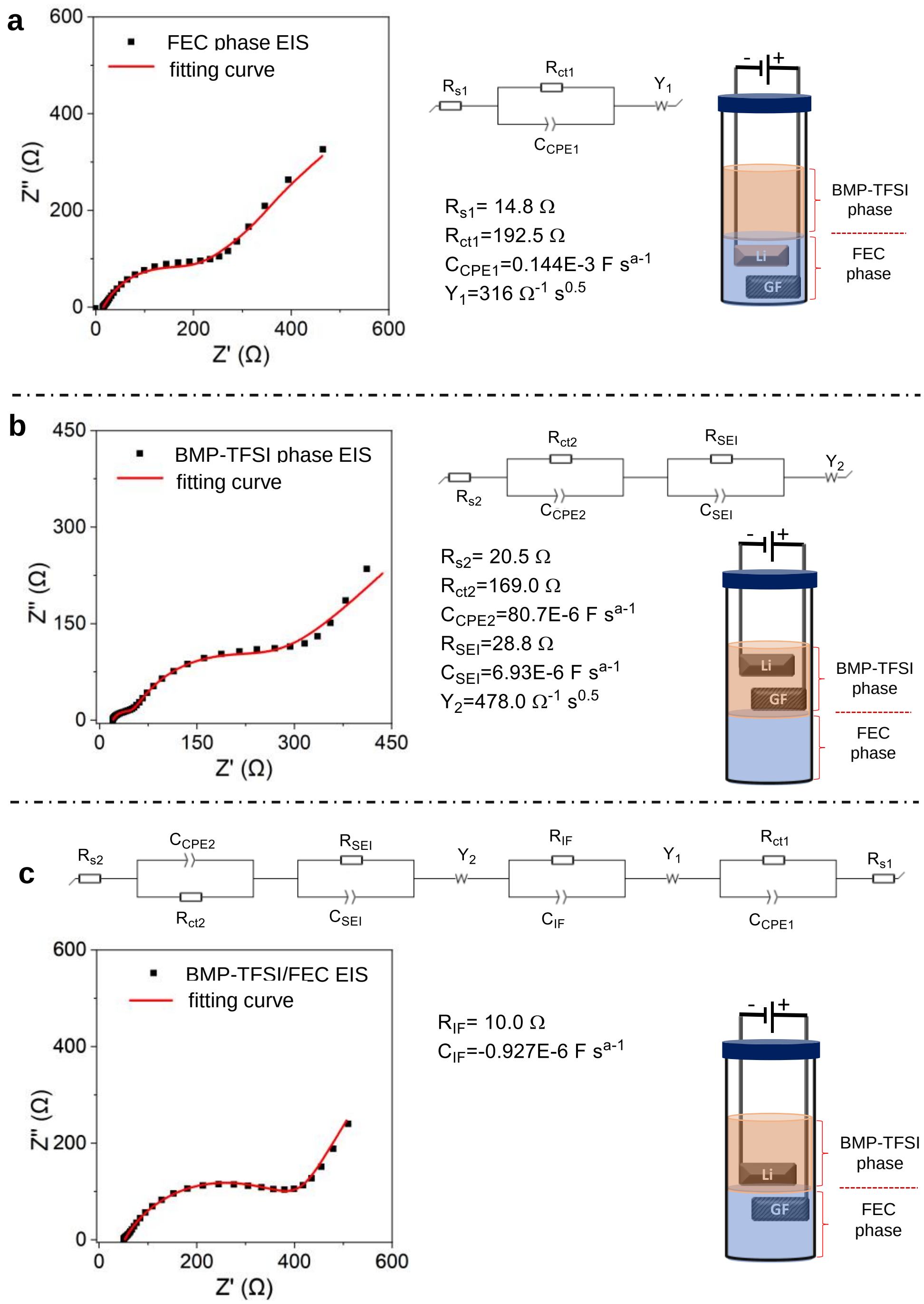

Supplementary Figure 4. The Nyquist plot, Nyquist fitting curve, and schematic diagram of the test setup of the (a) FEC/$LiClO_4$, (b) BMP-TFSI/LiTFSI, and (c) a nonaqueous biphasic system (NBS). The raw impedance data is reported as symbols and fitted data as lines. Electrochemical impedance spectroscopy was conducted to examine ion transport kinetics in different phases and at the liquid-liquid interface. The investigated systems include the FEC electrolyte, BMP-TFSI electrolyte, and the BMP-TFSI/FEC (liquid/liquid) system. The EIS experiments were carried out at 27°C.

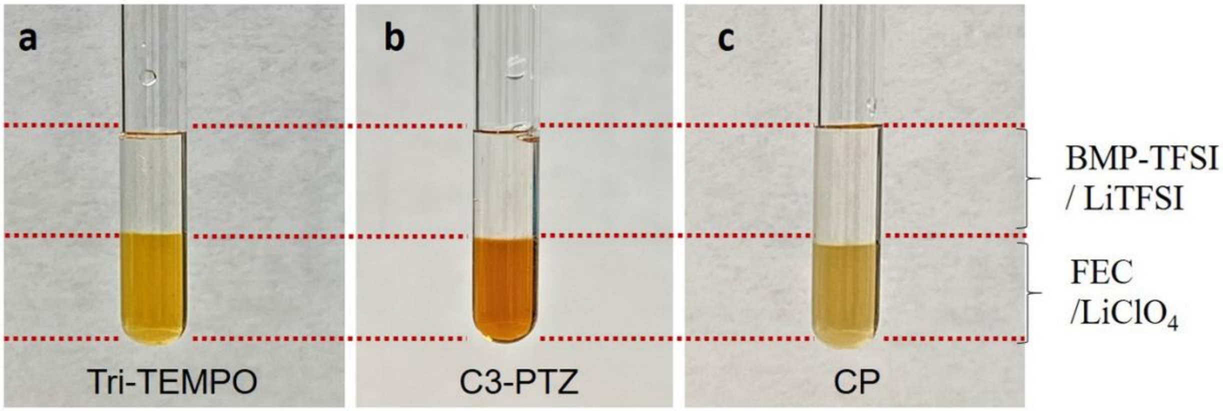

Supplementary Figure 5. Digital photographs illustrating a nonaqueous biphasic system (NBS) containing (a) Tri-TEMPO, (b) C3-PTZ, and (c) CP as redox-active catholytes. The catholytes (Tri-TEMPO, C3-PTZ, and CP) exhibit good solubility in the cathode electrolyte (FEC/LiClO4) while displaying minimal affinity towards the anode electrolyte (BMP-TFSI/LiTFSI), rendering them ideal candidates for utilization in the developed NBS.

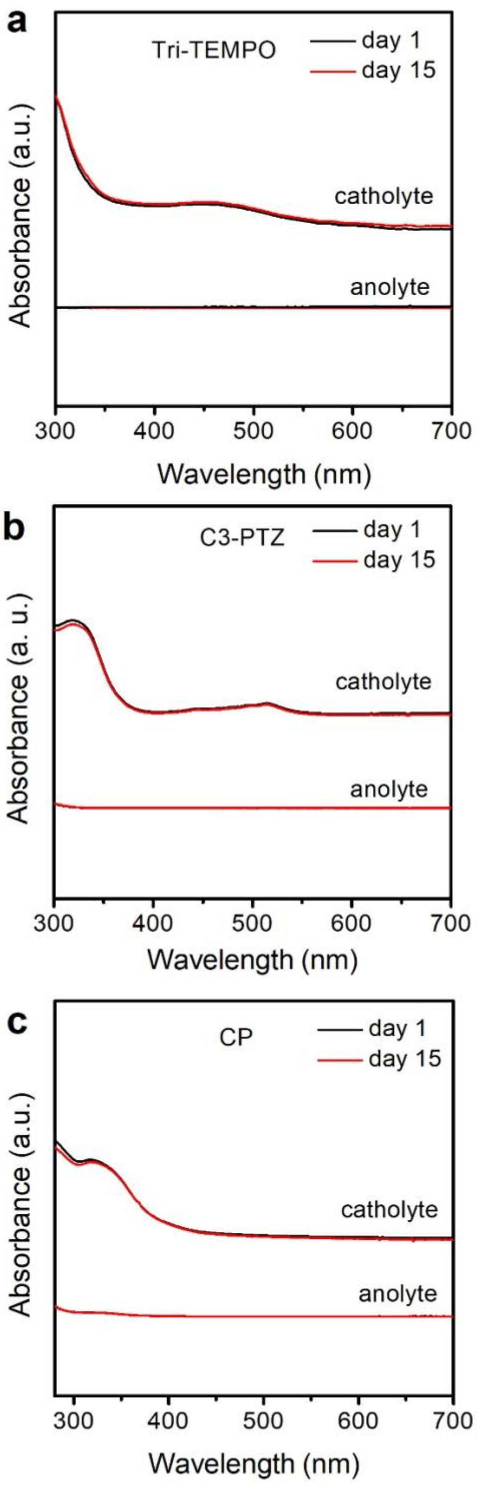

Supplementary Figure 6. UV-visible spectra comparison of the catholyte and anolyte in a nonaqueous biphasic system containing (a) 0.5 M Tri-TEMPO, (b) 0.5 M C3-PTZ, and (c) 0.5 M CP on day 1 and day 15. No significant change in the intensity of the spectra is observed before and after 15 days, indicating no evidence of redox active material crossover from the cathode electrolyte to the anode electrolyte.

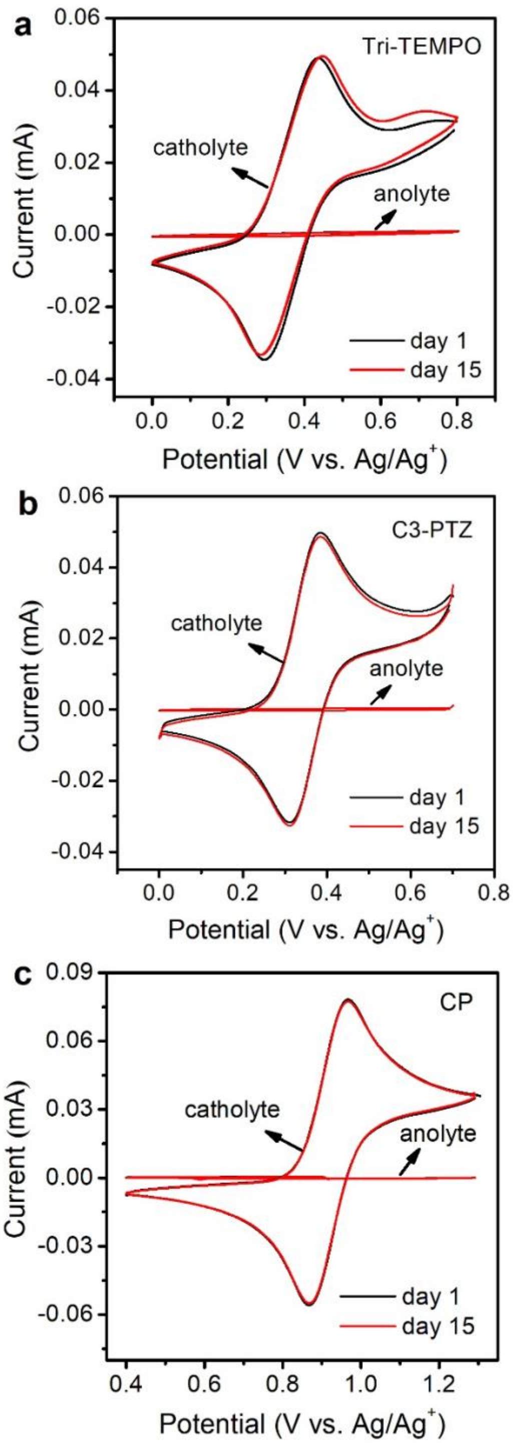

Supplementary Figure 7. Cyclic voltammograms comparison of the catholyte and anolyte in a nonaqueous biphasic system containing (a) 0.5 M Tri-TEMPO, (b) 0.5 M C3-PTZ, and (c) 0.5 M CP on day 1 and day 15 at a scan rate of 50 mV/s and temperature of 27°C. No significant change in the peak current is observed before and after 15 days, indicating no evidence of redox active material crossover from the cathode electrolyte to the anode electrolyte.

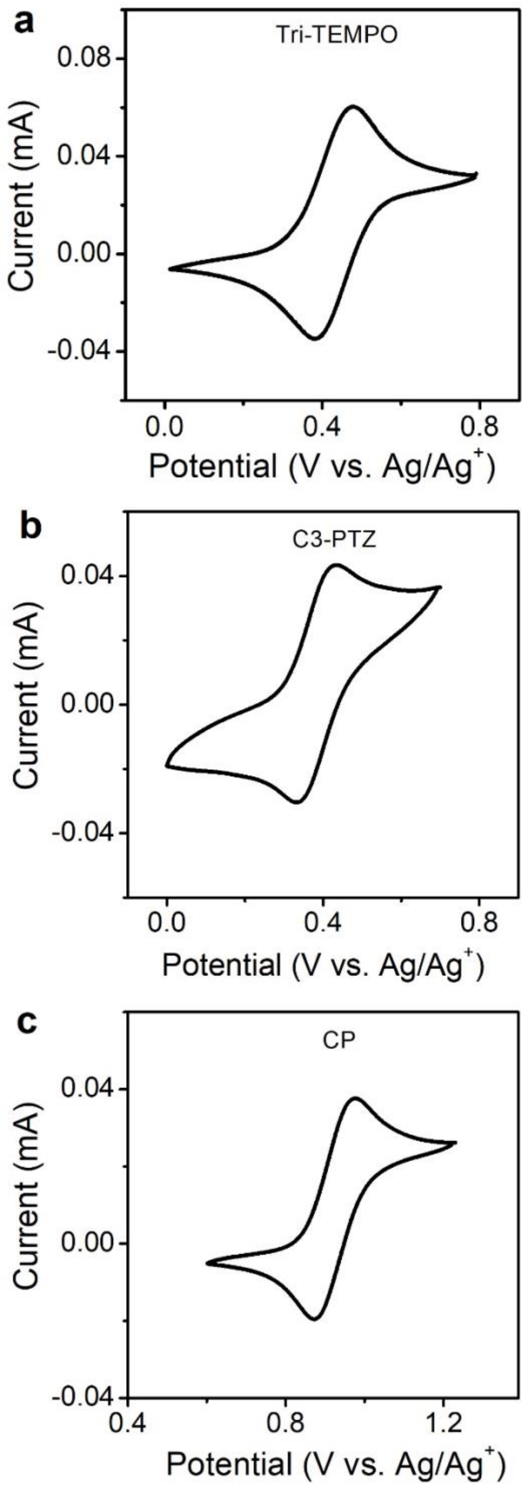

Supplementary Figure 8. Cyclic voltammograms of 5 mM (a) Tri-TEMPO, (b) C3-PTZ, and (c) CP in 0.1 M FEC/$LiClO_4$ solution. The measurements were performed at a scan rate of 5 mV/s and a temperature of 27°C.

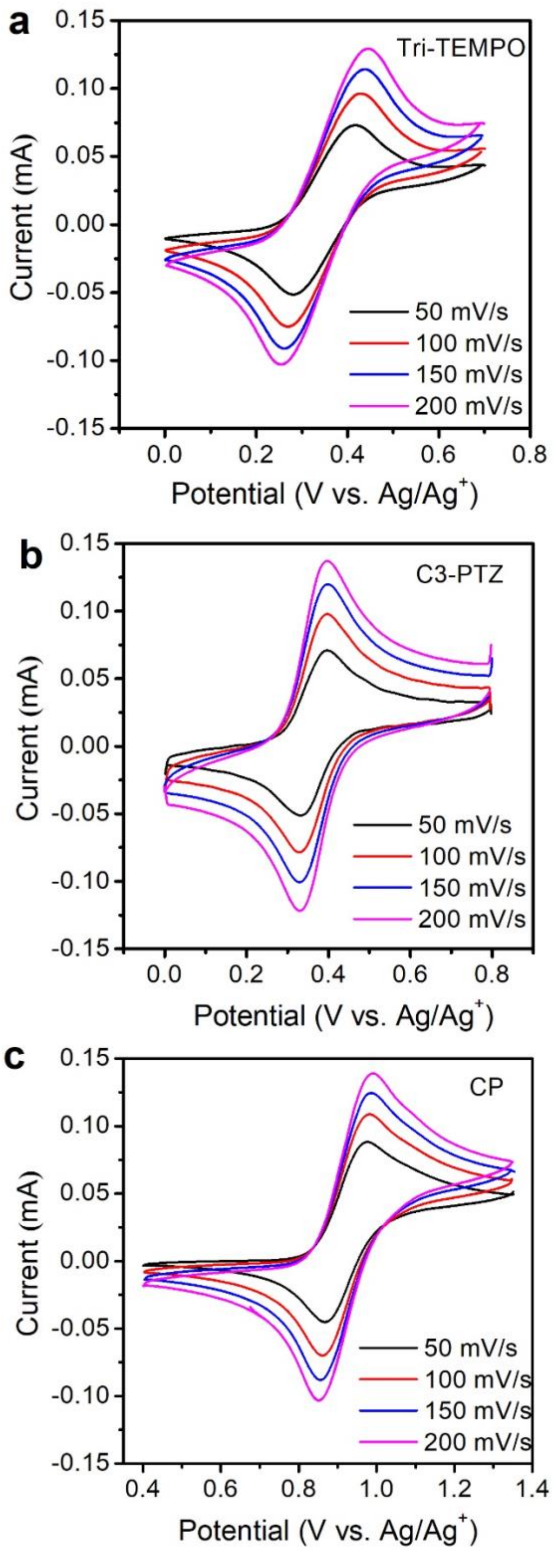

Supplementary Figure 9. Cyclic voltammograms of 5 mM (a) Tri-TEMPO, (b) C3-PTZ, and (c) CP in 0.1 M FEC/$LiClO_4$ solution. The measurements were carried out using scan rates ranging from 50 to 200 mV/s at a temperature of 27°C.

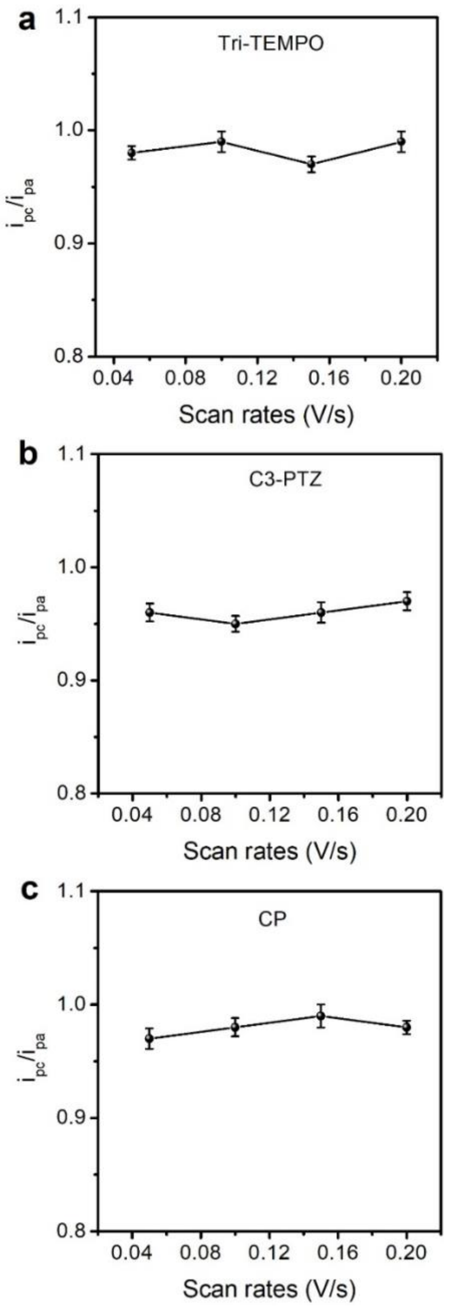

Supplementary Figure 10. The i_{pc}/i_{pa} curve of 5mM (a) Tri–TEMPO, (a) C3–PTZ, and (C) CP in 0.1 M FEC/$LiClO_4$ solution. The measurements were carried out using scan rates ranging from 50 to 200 mV/s at a temperature of 27°C. The error bars in each figure represent the standard deviation (±SD) (calculated from an average of three data points used for each figure).

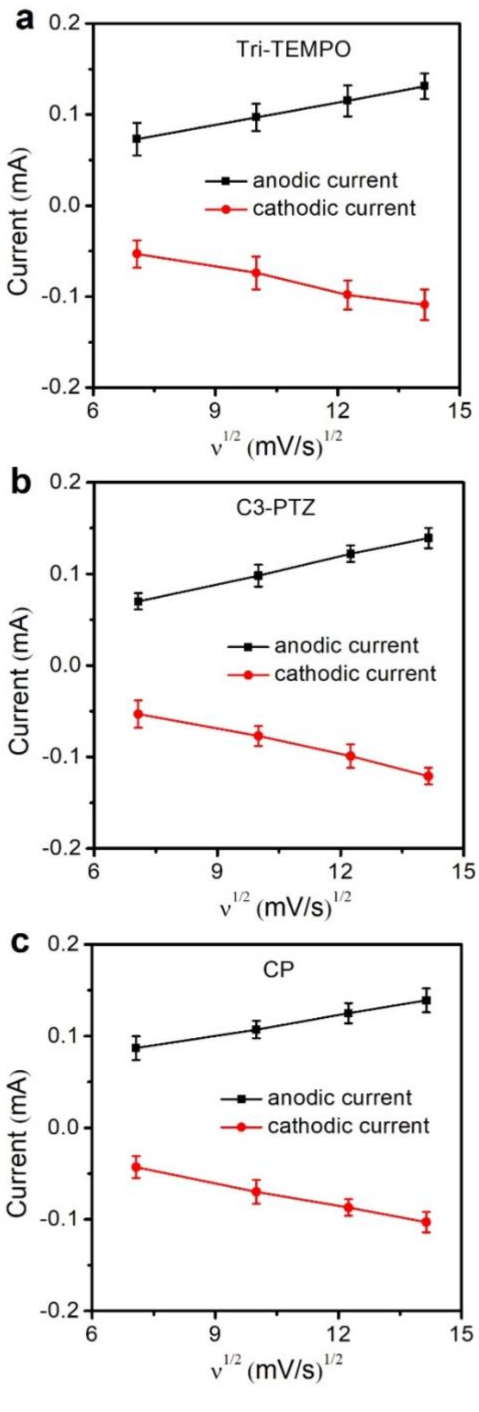

Supplementary Figure 11. Current vs. square root curve of scan rates curves of 5 mM (a) Tri-TEMPO, (b) C3-PTZ, and (c) CP in 0.1 M FEC/$LiClO_4$ solution. The measurements were carried out using scan rates ranging from 50 to 200 mV/s at a temperature of 27°C. The error bars in each figure represent the standard deviation (±SD) (calculated from an average of three data points used for each figure).

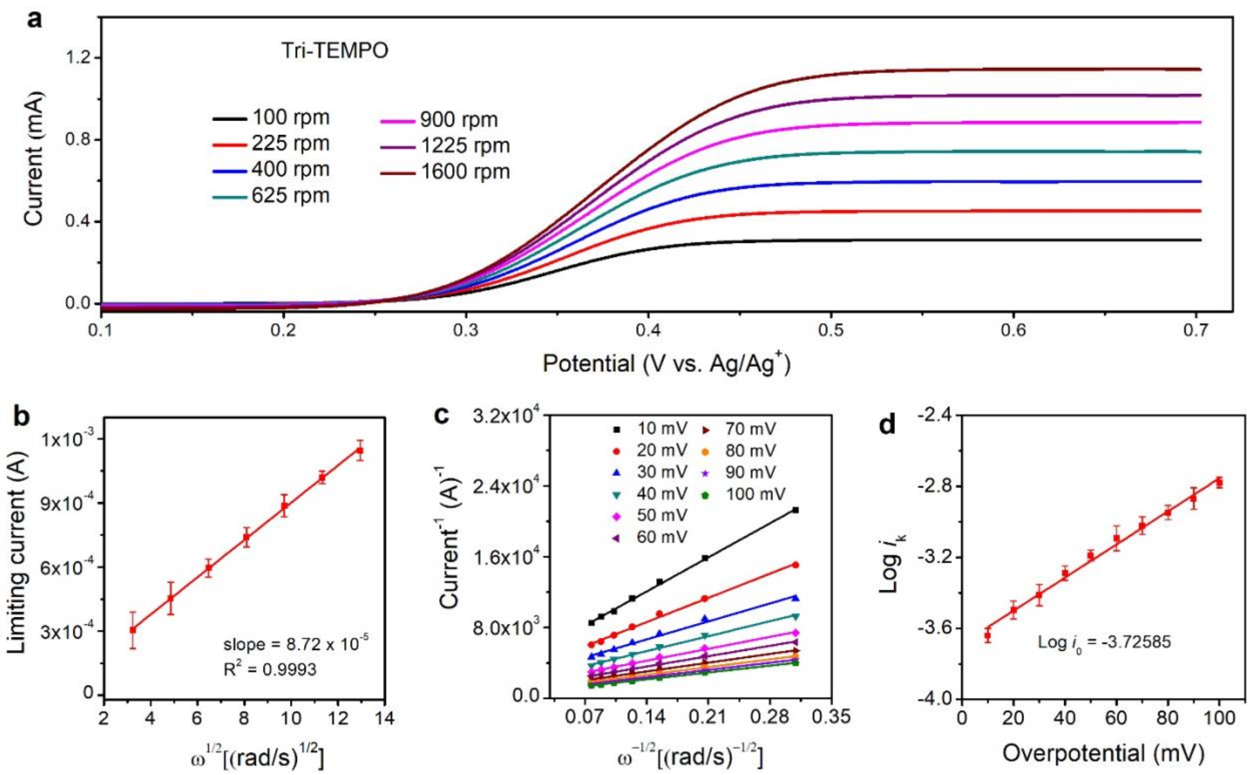

Supplementary Figure 12. RDE studies of Tri-TEMPO using electrolyte solutions containing 0.5 mM redox material in 0.1 M FEC/$LiClO_4$. The error bars in each figure represent the standard deviation (±SD) (calculated from an average of three data points used for each figure).

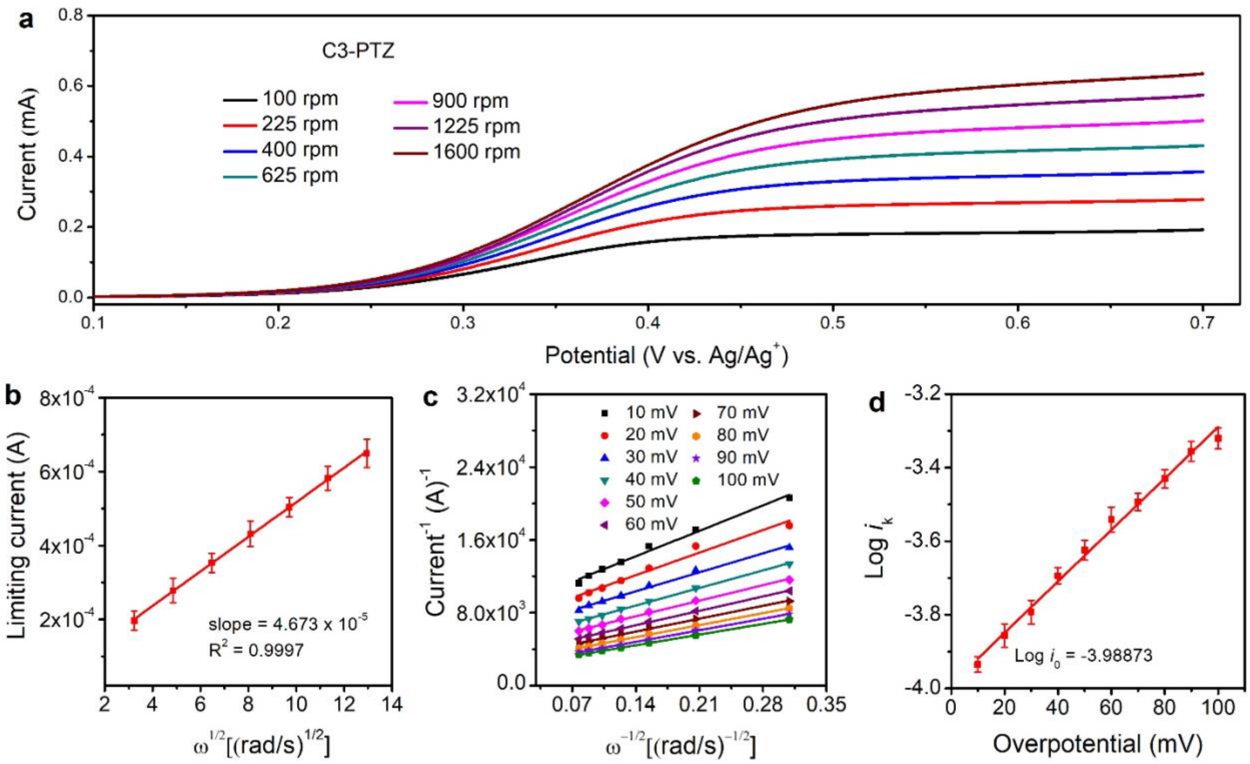

Supplementary Figure 13. RDE studies of C3-PTZ using electrolyte solutions containing 0.5 mM redox material in 0.1 M FEC/$LiClO_4$. The error bars in each figure represent the standard deviation (±SD) (calculated from an average of three data points used for each figure).

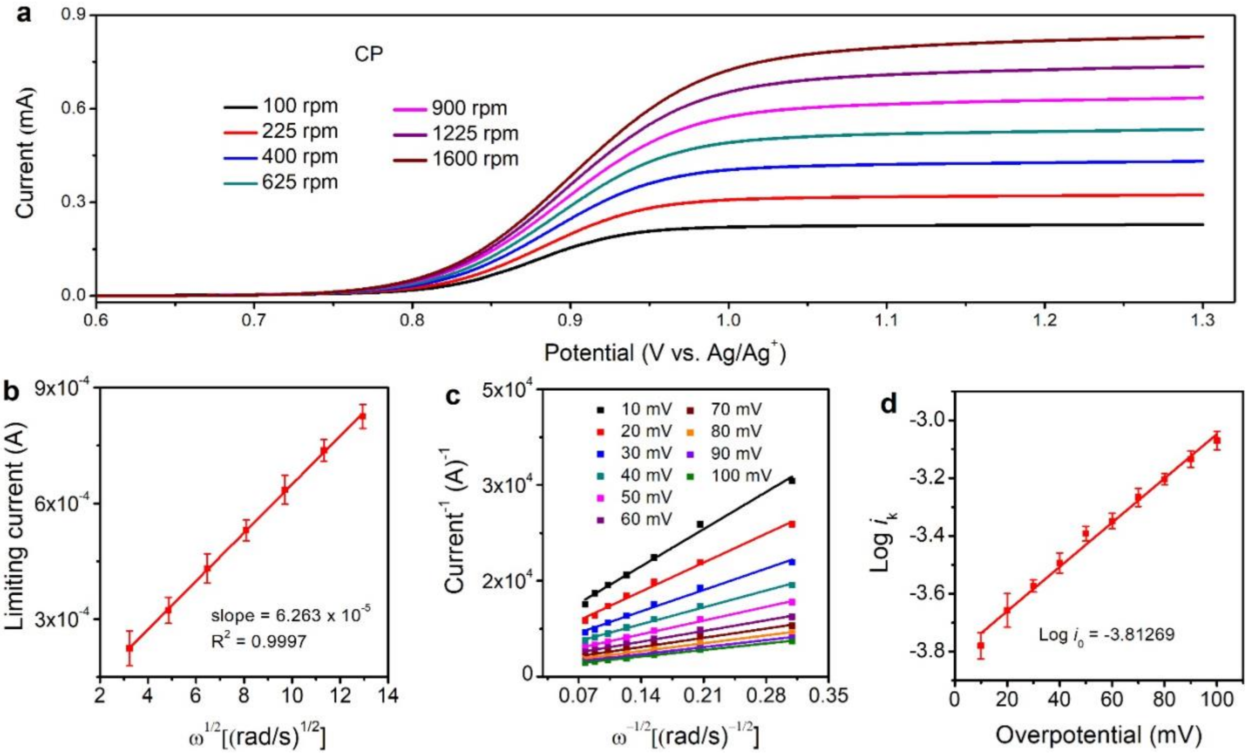

Supplementary Figure 14. RDE studies of CP using electrolyte solutions containing 0.5 mM redox material in 0.1 M FEC/$LiClO_4$. The error bars in each figure represent the standard deviation (±SD) (calculated from an average of three data points used for each figure).

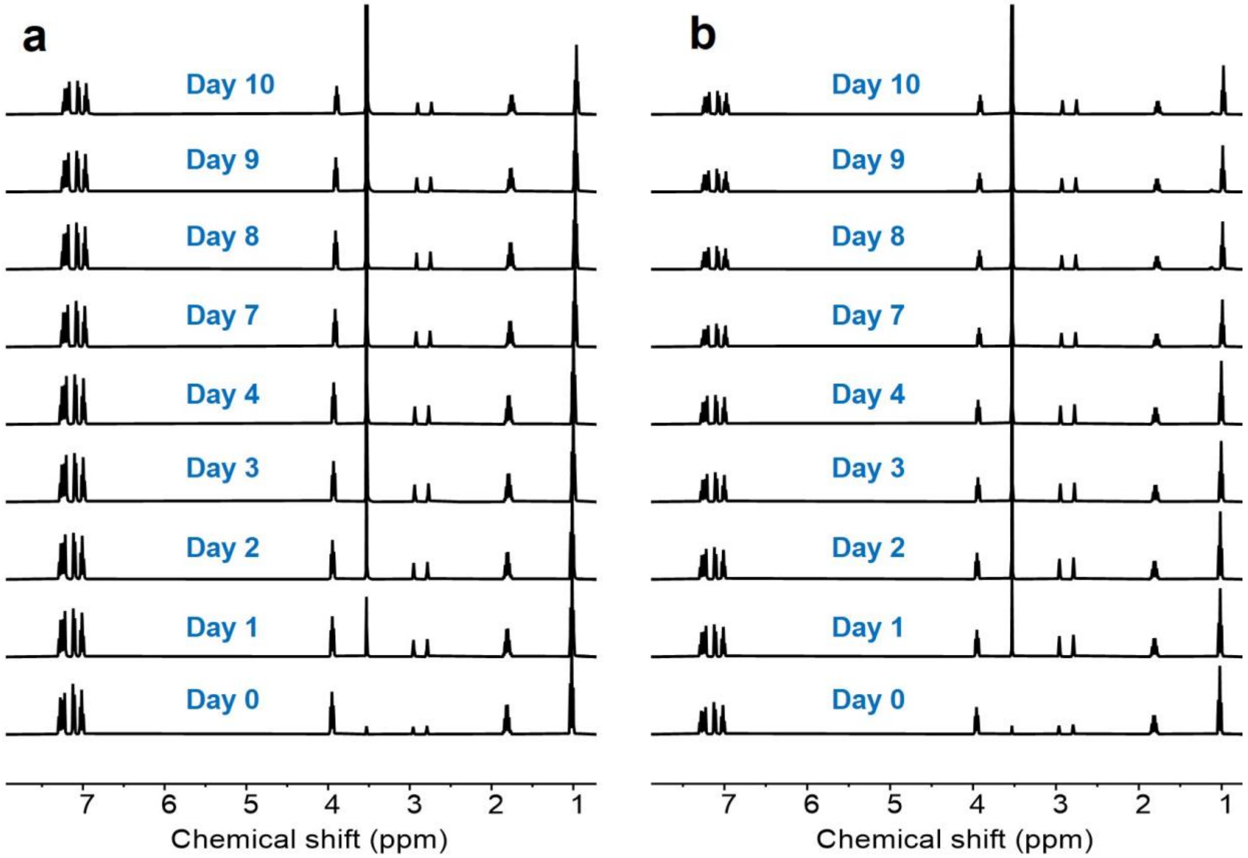

Supplementary Figure 15. ^{1}H NMR spectra of C3-PTZ at (a) 25°C and (b) 60°C.

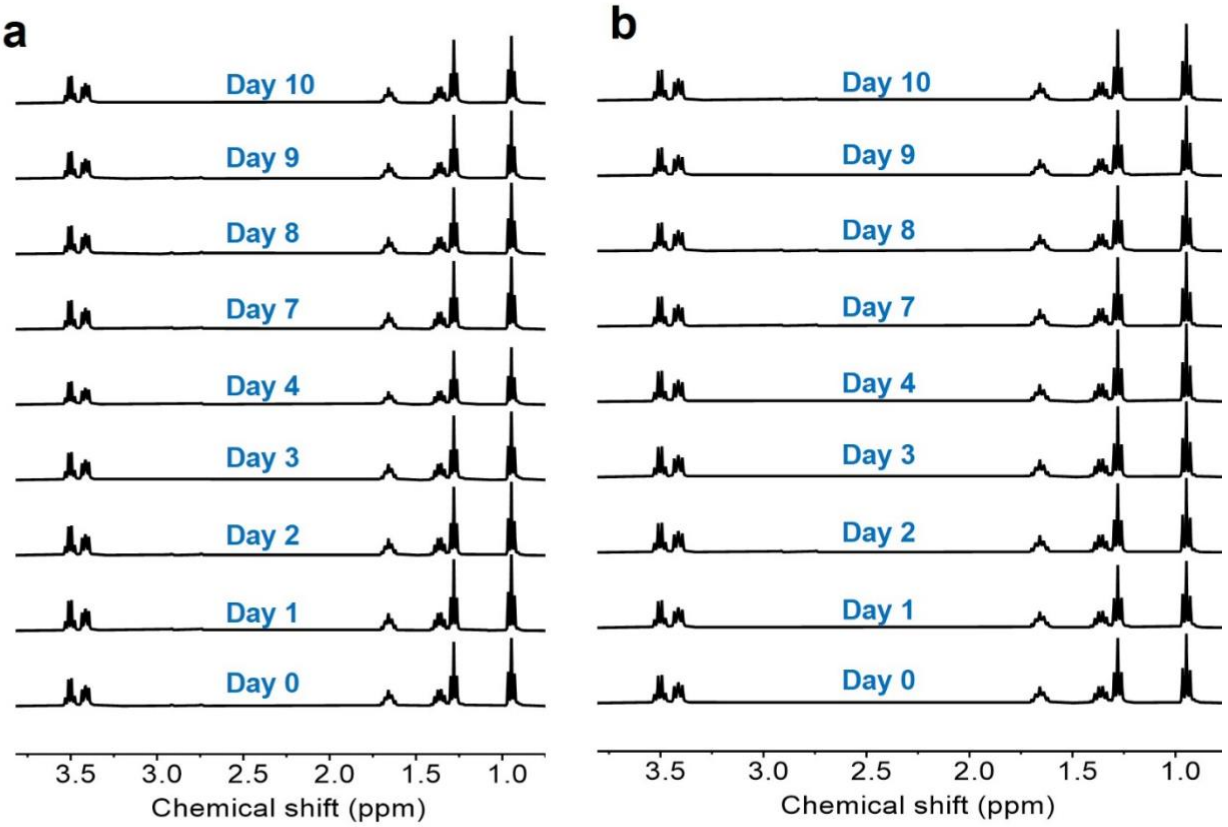

Supplementary Figure 16. ^{1}H NMR spectra of **CP** at (a) 25°C and (b) 60°C.

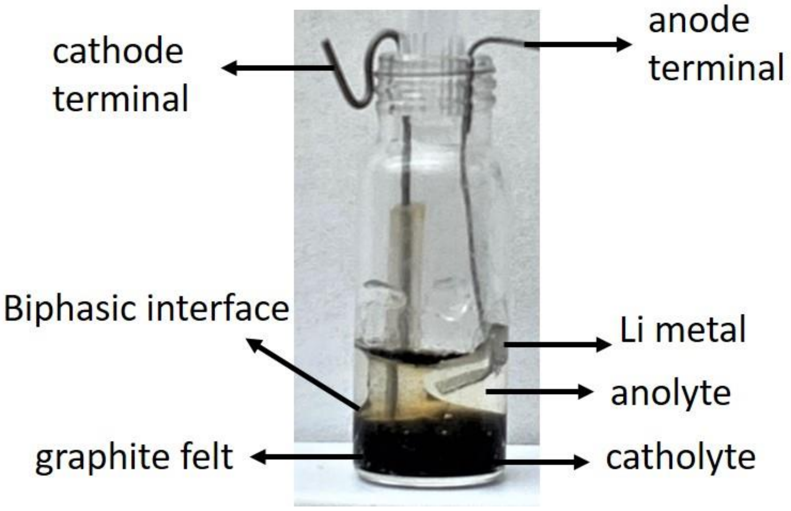

Supplementary Figure 17. Digital photograph illustrating the various components of a nonaqueous biphasic static battery system.

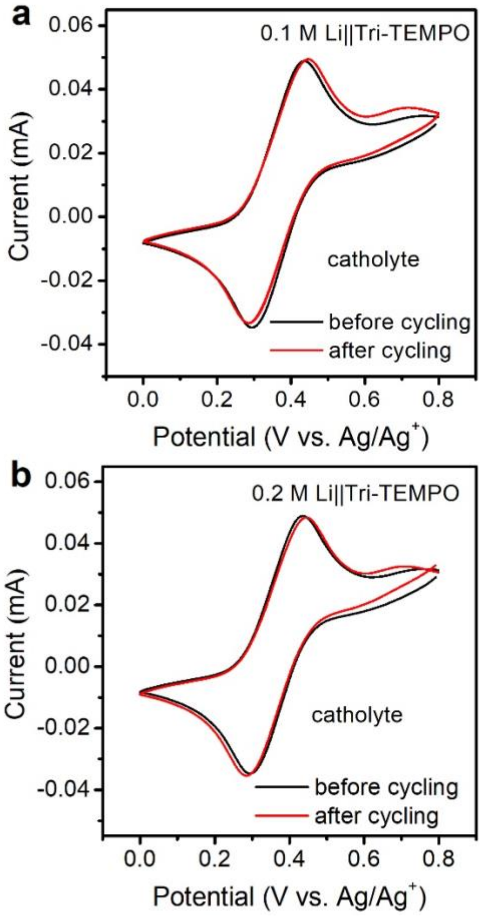

Supplementary Figure 18. Cyclic voltammograms of (a) 0.1 M and (b) 0.2 M Li||Tri-TEMPO battery catholytes before and after 100 charge/discharge cycles. The measurements were carried out at a scan rate of 50 mV/s at a temperature of 27°C.

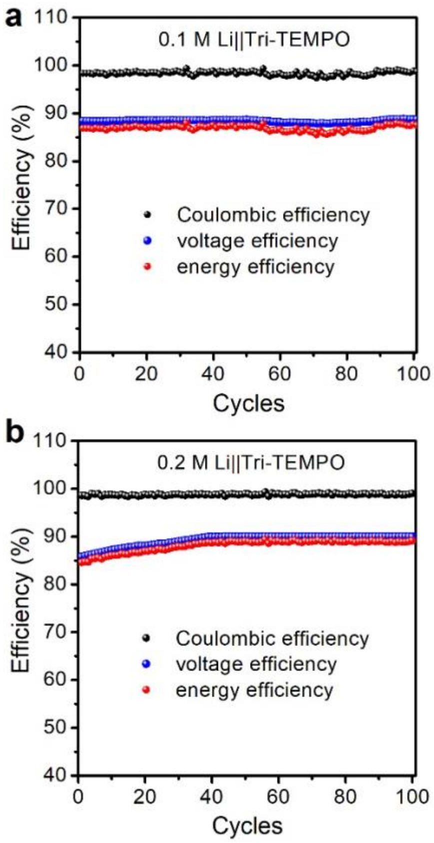

Supplementary Figure 19. Variation in Coulombic efficiency, voltage efficiency, and energy efficiency of (a) 0.1 M and (b) 0.2 M Li||Tri-TEMPO static battery for 100 charge/discharge cycle. The charge/discharge measurements were carried at the current density of 1 mA/cm^2 at a temperature of 27°C.

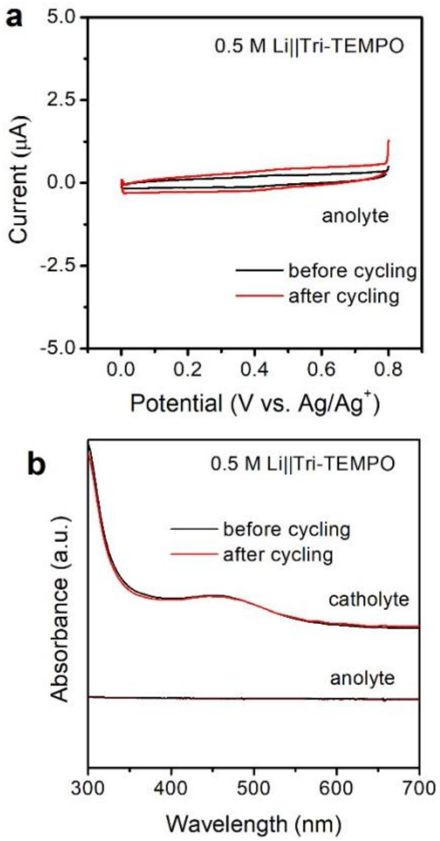

Supplementary Figure 20. (a) Cyclic voltammograms (anolyte) and (b) UV-visible spectra of the 0.5 M Li||Tri-TEMPO static battery electrolytes before and after 100 charge/discharge cycles. The CV measurements were carried at a scan rate of 50 mV/s at a temperature of 27°C.

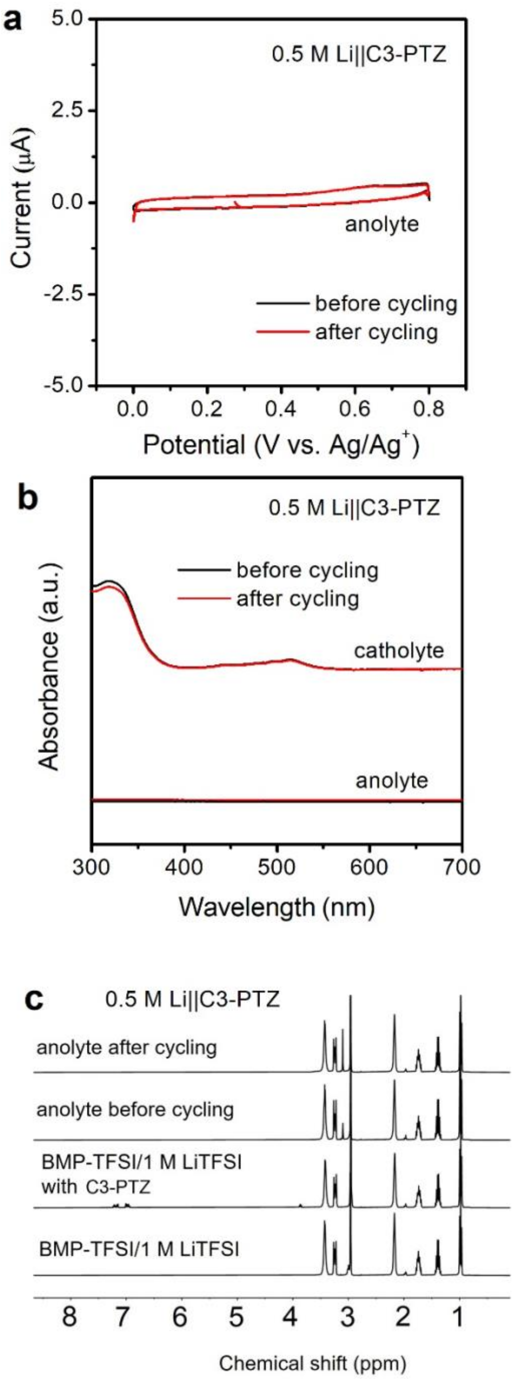

Supplementary Figure 21. (a) Cyclic voltammograms (anolyte), (b) UV-visible spectra, and (c) Stacked ^{1}H NMR spectra collected for a pure BMP-TFSI (bottom), pure C3-PTZ with BMP-TFSI (2nd spectrum from the bottom), BMP-TFSI anolyte collected before (2nd spectrum from the top) and after (top) 100 charge/discharge cycles. All NMR samples contain 1 M LiTFSI electrolytes. The CV measurements were carried at a scan rate of 50 mV/s at a temperature of 27°C.

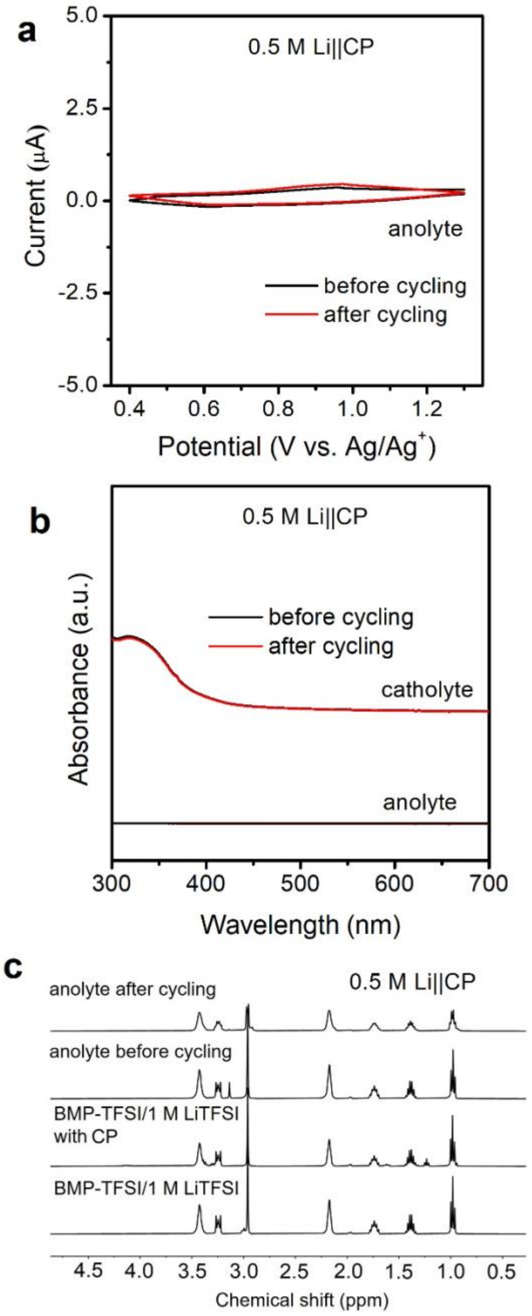

Supplementary Figure 22. (a) Cyclic voltammograms (anolyte), (b) UV-visible spectra, and (c) Stacked ^{1}H NMR spectra collected for a pure BMP-TFSI (bottom), pure CP with BMP-TFSI (2nd spectrum from the bottom), BMP-TFSI anolyte collected before (2nd spectrum from the top) and after (top) 100 charge/discharge cycles. All NMR samples contain 1 M LiTFSI electrolytes. The CV measurements were carried at a scan rate of 50 mV/s at a temperature of 27°C.

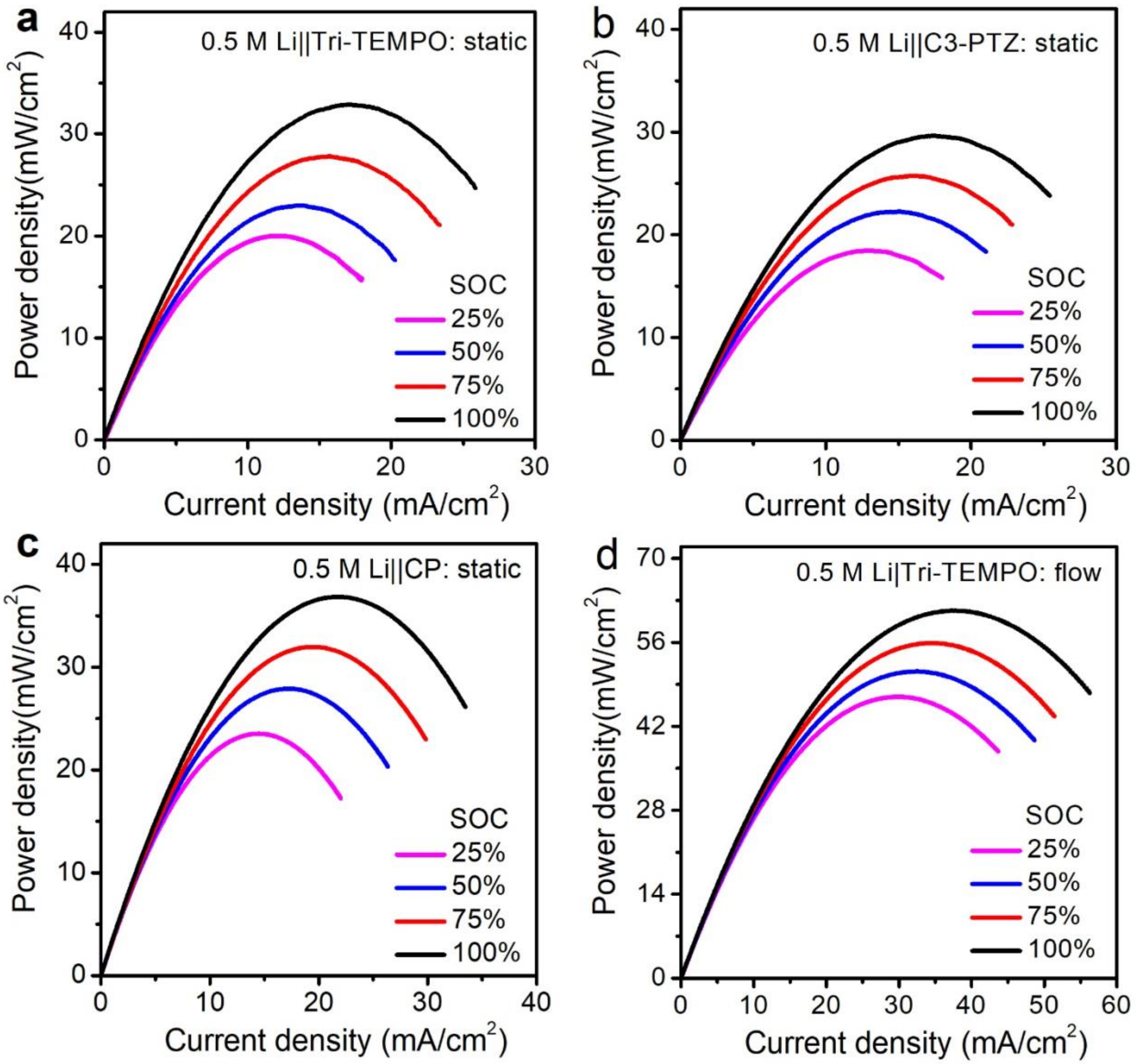

Supplementary Figure 23. Polarization curve at different states-of-charge (SOC) of (a) 0.5 M Li||Tri-TEMPO, (b) 0.5 M Li||C3-PTZ, (c) 0.5 M Li||CP batteries under static conditions, and (d) 0.5 M Li||Tri-TEMPO battery under flow conditions. The power density measurements were performed at a temperature of 27°C.

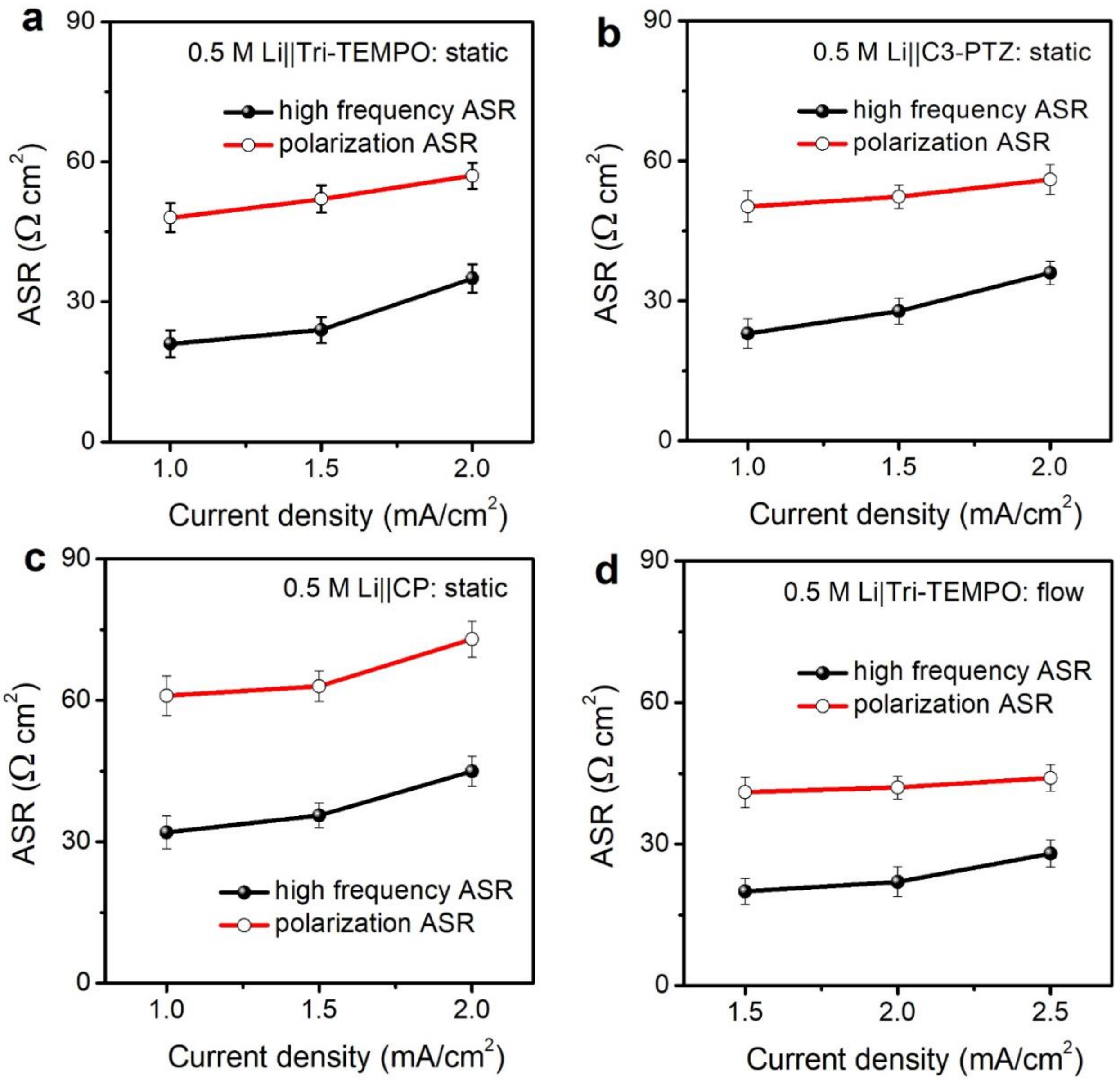

Supplementary Figure 24. Area-specific resistance (ASR) at different operating current densities of (a) 0.5 M Li||Tri-TEMPO, (b) 0.5 M Li||C3-PTZ, (c) 0.5 M Li||CP battery under static conditions, and (d) 0.5 M Li||Tri-TEMPO battery under flow conditions. The error bars in each figure represent the standard deviation (±SD) (calculated from an average of three data points used for each figure).

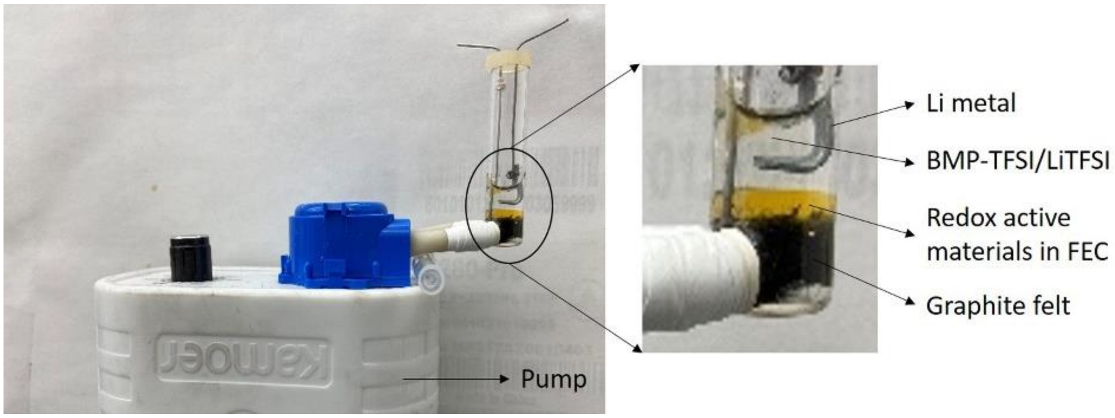

Supplementary Figure 25. Photograph of the setup of a membrane-free nonaqueous biphasic flow battery.

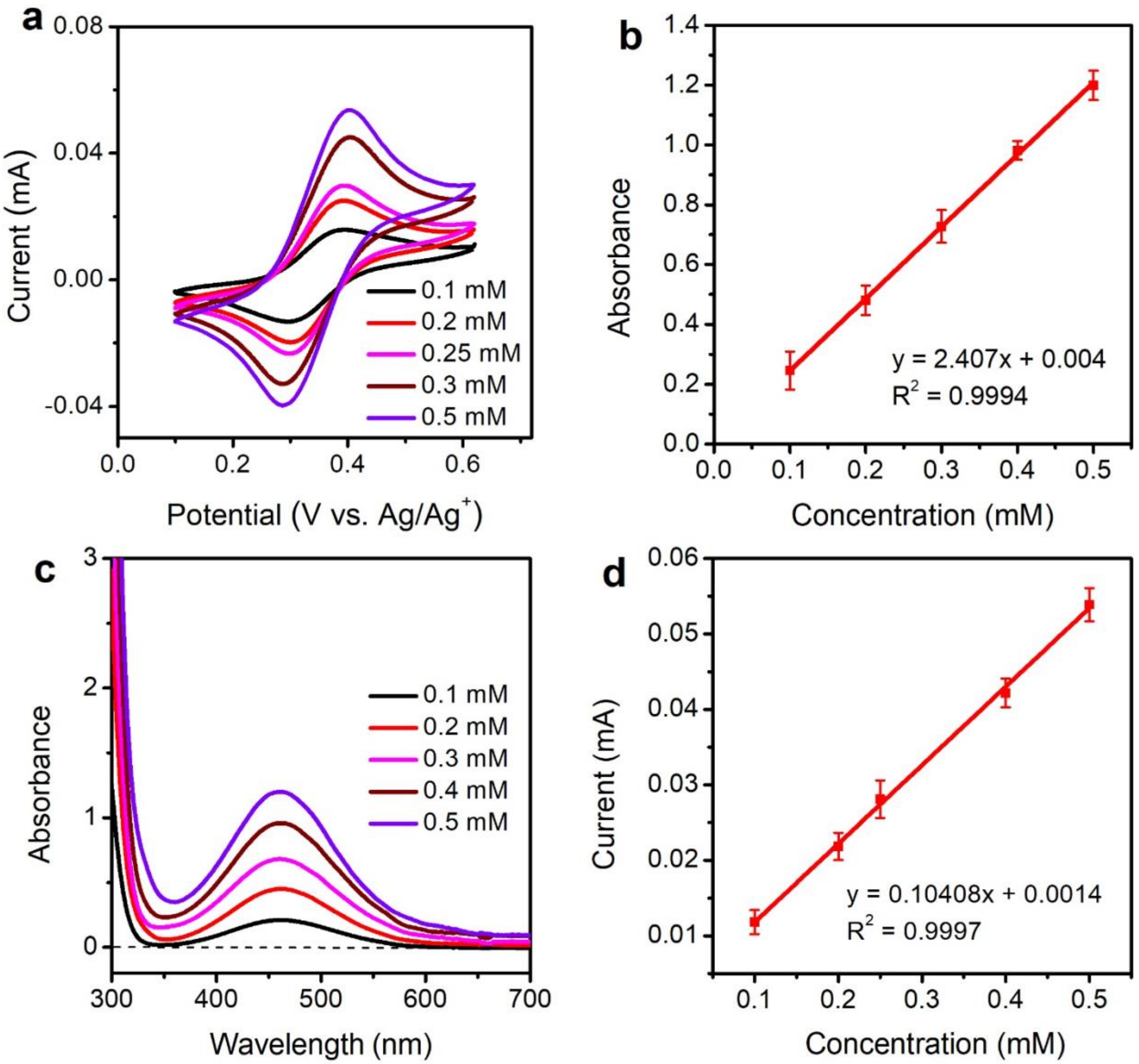

Supplementary Figure 26. (a and b) CV calibration curve and (c and d) UV-visible calibration curve to measure the crossover of redox materials across the interface in the 0.5 M Li||Tri-TEMPO flow battery.

Supplementary Table 1. Development of nonaqueous biphasic systems.

**Catholyte →** / **Anolyte ↓**	THF	FEC	TGDME	DEC	PC	BTF (80%) + TEGDME (20%)
	**With no salt**					
EMI- TFSI	**M**	**M**	**PM**	**M**	**M**	**M**
NFTTS	**M**	**IM**	**M**	**IM**	**IM**	**M**
$EMI-BF_4$	**M**	**PM**	**PM**	**M**	**M**	**PM**
PMP-TFSI	**M**	**PM**	**PM**	**PM**	**M**	**M**
BMP-TFSI	**M**	**PM**	**PM**	**PM**	**M**	**M**
	**With 0.5 M salt**					
EMI- TFSI	**PM** (1 M LiTFSI in EMI-TFSI) (0.5 M $LiClO_4$ in THF)	**PM** (1 M LiTFSI in EMI-TFSI) (0.5 M $LiClO_4$ in FEC)	**PM** (1 M LiTFSI in EMI-TFSI) (0.5 M $LiClO_4$ in FEC)	**PM** (1 M LiTFSI in EMI-TFSI) (0.5 M $LiClO_4$ + 1 M $TBABF_4$ in DEC)	**M** (1 M $LiPF_6$ in EMI-TFSI) (0.5 M $LiClO_4$ PC)	**M** (0.5 M $TBABF_4$ in both)
NFTTS	**M** (0.5 M LiTFSI in both)	**IM** (no salt)	**PM** (0.5 M LiTFSI in both)	**IM** (no salt)	**IM** (no salt)	**PM** (0.5 M LiTFSI in both)
$EMI-BF_4$	**PM** (0.5 M $TBABF_4$ in both)	**PM** (1 M LiTFSI in $EMI-BF_4$) (0.5 M $LiClO_4$ in FEC)	**PM** (1.5 M $TBAPF_6$ in $EMI-BF_4$) (0.5 M $LiPF_6$ in TEGDME)	**PM** (1 M $LiPF_6$ in $EMI-BF_4$) (0.5 M $LiClO_4$ in DEC)	**PM** (1 M $TBAPF_6$ + 1 M $LiPF_6$ in $EMI-BF_4$) (0.5 M $LiClO_4$ PC)	**PM** (0.5 M $TBABF_4$ in (BTF+TEGDME) mixture
PMP-TFSI	**PM** (1 M $LiPF_6$ in PMP-TFSI) (0.5 M $TBAF_6$ in THF)	**PM** (1 M LiTFSI in PMP-TFSI) (0.5 M $LiClO_4$ in FEC)	**PM** (1 M LiTFSI in PMP-TFSI) (0.5 M $LiClO_4$ in TEGDME)	**PM** (1 M $LiPF_6$ in PMP-TFSI) (0.5 M $LiClO_4$ in DEC)	**PM** (1 M $TBAPF_6$ + 1 M $LiPF_6$ in PMP-TFSI) (0.5 M $LiClO_4$ PC)	**PM** (0.5 M $TBABF_4$ in both)
BMP-TFSI	**PM** (1 M $LiPF_6$ in BMP-TFSI) (0.5 M $TBAF_6$ in THF)	**PM** (1 M LiTFSI in BMP-TFSI) (0.5 M $LiClO_4$ in FEC)	**PM** (1 M LiTFSI in BMP-TFSI) (0.5 M $LiClO_4$ in TEGDME)	**PM** (1 M $LiPF_6$ in BMP-TFSI) (0.5 M $LiClO_4$ in DEC)	**PM** (1 M $TBAPF_6$ + 1 M $LiPF_6$ in BMP-TFSI) (0.5 M $LiClO_4$ PC)	**M** (0.5 M $TBABF_4$ in both)

**With 1.0 M salt**						
EMI- TFSI	**PM**<br>(1 M LiTFSI in EMI-TFSI)<br>(1 M $LiClO_4$ in THF)	**PM**<br>(1 M LiTFSI in EMI-TFSI)<br>(1 M $LiClO_4$ in FEC)	**PM**<br>(1 M LiTFSI in EMI-TFSI)<br>(1 M $LiClO_4$ in FEC)	**PM**<br>(1 M LiTFSI in EMI-TFSI)<br>(1 M $LiClO_4$ + 1 M $TBABF_4$ in DEC)	**M**<br>(1 M $LiPF_6$ in EMI-TFSI)<br>(1 M $LiClO_4$ PC)	**M**<br>(1 M $TBABF_4$ in both)
NFTTS	**M**<br>(1 M LiTFSI in both)	**IM**<br>(no salt)	**IM**<br>(1 M LiTFSI in both)	**IM**<br>(no salt)	**IM**<br>(no salt)	**IM**<br>(1 M LiTFSI in both)
EMI-BF_4	**PM**<br>(1 M $TBABF_4$ in both)	**PM**<br>(1 M LiTFSI in EMI-BF_4)<br>(1 M $LiClO_4$ in FEC)	**IM**<br>(1.5 M $TBAPF_6$ in EMI-BF_4)<br>(1 M $LiPF_6$ in TEGDME)	**PM**<br>(1 M $LiPF_6$ in EMI-BF_4)<br>(1 M $LiClO_4$ in DEC)	**PM**<br>(1 M $TBAPF_6$ + 1 M $LiPF_6$ in EMI-BF_4)<br>(1 M $LiClO_4$ PC)	**IM**<br>(1 M $TBABF_4$ in (BTF+TEGDME) mixture
PMP-TFSI	**PM**<br>(1 M $LiPF_6$ in PMP-TFSI)<br>(1 M $TBAF_6$ in THF)	**PM**<br>(1 M LiTFSI in PMP-TFSI)<br>(1 M $LiClO_4$ in FEC)	**PM**<br>(1 M LiTFSI in PMP-TFSI)<br>(1 M $LiClO_4$ in TEGDME)	**PM**<br>(1 M $LiPF_6$ in PMP-TFSI)<br>(1 M $LiClO_4$ in DEC)	**PM**<br>(1 M $TBAPF_6$ + 1 M $LiPF_6$ in PMP-TFSI)<br>(1 M $LiClO_4$ PC)	**PM**<br>(1 M $TBABF_4$ in both)
BMP-TFSI	**PM**<br>(1 M $LiPF_6$ in BMP-TFSI)<br>(1 M $TBAF_6$ in THF)	**PM**<br>(1 M LiTFSI in BMP-TFSI)<br>(1 M $LiClO_4$ in FEC)	**PM**<br>(1 M LiTFSI in BMP-TFSI)<br>(1 M $LiClO_4$ in TEGDME)	**PM**<br>(1 M $LiPF_6$ in BMP-TFSI)<br>(1 M $LiClO_4$ in DEC)	**PM**<br>(1 M $TBAPF_6$ + 1 M $LiPF_6$ in BMP-TFSI)<br>(1 M $LiClO_4$ PC)	**M**<br>(1 M $TBABF_4$ in both)
**With 1.5 M Salt**						
EMI- TFSI	**PM**<br>(1 M LiTFSI in EMI-TFSI)<br>(1.5 M $LiClO_4$ in THF)	**PM**<br>(1 M LiTFSI in EMI-TFSI)<br>(1.5 M $LiClO_4$ in FEC)	**PM**<br>(1 M LiTFSI in EMI-TFSI)<br>(1.5 M $LiClO_4$ in FEC)	**PM**<br>(1 M LiTFSI in EMI-TFSI)<br>(1.5 M $LiClO_4$ + 1 M $TBABF_4$ in DEC)	**M**<br>(1 M $LiPF_6$ in EMI-TFSI)<br>(1.5 M $LiClO_4$ PC)	**M**<br>(1.5 M $TBABF_4$ in both)
NFTTS	**M**<br>(1.5 M LiTFSI in both)	**IM**<br>(no salt)	**PM**<br>(1.5 M LiTFSI in both)	**IM**<br>(no salt)	**IM**<br>(no salt)	**PM**<br>(1.5 M LiTFSI in both)
EMI-BF_4	**PM**<br>(1.5 M $TBABF_4$ in both)	**PM**<br>(1 M LiTFSI in EMI-BF_4)<br>(1.5 M $LiClO_4$ in FEC)	**PM**<br>(1.5 M $TBAPF_6$ in EMI-BF_4)	**PM**<br>(1 M $LiPF_6$ in EMI-BF_4)<br>(1.5 M $LiClO_4$ in DEC)	**PM**<br>(1 M $TBAPF_6$ + 1 M $LiPF_6$ in EMI-BF_4)<br>(1.5 M $LiClO_4$ PC)	**IM**<br>(1.5 M $TBABF_4$ in (BTF+TEGDME) mixture

			(1.5 M $LiPF_6$ in TEGDME)			(Not stable for the long term)
PMP-TFSI	**PM** (1 M $LiPF_6$ in PMP-TFSI) (1.5 M $TBAF_6$ in THF)	**PM** (1 M LiTFSI in PMP-TFSI) (1.5 M $LiClO_4$ in FEC)	**PM** (1 M LiTFSI in PMP-TFSI) (1.5 M $LiClO_4$ in TEGDME)	**PM** (1 M $LiPF_6$ in PMP-TFSI) (1.5 M $LiClO_4$ in DEC)	**PM** (1 M $TBAPF_6$ + 1 M $LiPF_6$ in PMP-TFSI) (1.5 M $LiClO_4$ PC)	**PM** (1 M $TBABF_4$ in both)
BMP-TFSI	**PM** (1 M $LiPF_6$ in BMP-TFSI) (1.5 M $TBAF_6$ in THF)	**IM** (1 M LiTFSI in BMP-TFSI) (1.5 M $LiClO_4$ in FEC)	**PM** (1 M LiTFSI in BMP-TFSI) (1.5 M $LiClO_4$ in TEGDME)	**PM** (1 M $LiPF_6$ in BMP-TFSI) (1.5 M $LiClO_4$ in DEC)	**PM** (1 M $TBAPF_6$ + 1 M $LiPF_6$ in BMP-TFSI) (1.5 M $LiClO_4$ PC)	**M** (1.5 M $TBABF_4$ in both)

M: Miscible, PM: Partially Miscible, IM: immiscible

Supplementary Table 2. Screening of cathode materials for FEC/BMP-TFSI biphasic system.

	**C3-PTZ**	**C8-PTZ**	**C18-PTZ**	**PEG3-PTZ**	**PEG12-PTZ**	**PEG3-TTF**	**Tri-TEMPO**	**CP**	**TEMPO**	**PEG12-AQ**	**Fc**
**1 M LiTFSI/BMP-TFSI (anolyte)**	NS	PS	PS	S	S	S	NS	NS	PS	PS	S
**1.5 M $LiClO_4$/FEC (catholyte)**	S	S	S	S	S	S	S	S	S	S	S

NS: Not soluble, **PS**: Poor solubility (solubility < 0.1 M), **S**: Soluble (solubility > 0.5 M)

Supplementary Table 3. The equivalent circuit fitting data and the corresponding fitting error for EIS.

	fitting data	fitting error
R_{s1}	14.8 Ω	0.469 Ω
R_{ct1}	193 Ω	10.2 Ω
C_{CPE1}	0.144E-3 F s^{a-1}	1.66E-5 F s^{a-1}
Y_1	316 W^{-1} $s^{0.5}$	19.2 W^{-1} $s^{0.5}$
R_{s2}	20.5 Ω	0.407 Ω
R_{ct2}	169 Ω	3.80 Ω
C_{CPE2}	80.7E-6 F s^{a-1}	5.71E-7 F s^{a-1}
R_{SEI}	28.8 Ω	0.453 Ω
C_{SEI}	6.93E-6 F s^{a-1}	3.10E-7 F s^{a-1}
Y_2	478 W^{-1} $s^{0.5}$	15.2 W^{-1} $s^{0.5}$
R_{IF}	10.0 Ω	0.156 Ω
C_{IF}	-0.927E-6 F s^{a-1}	2.275E-8 F s^{a-1}

Supplementary Table 4. Performance comparison of liquid/liquid biphasic membrane-free batteries.

Batteries	Solvent	Concentration of RMs	Current density (mA/cm^2)	OCV	Energy Density (Wh/L)		Cycles	CE (%)	CR (%)	Supplementary References
Li‖Tri-TEMPO (static)	BMP-TFSI|FEC	0.5 M	1	3.53	34[a]	48[b]	100	96	98	This Work
Li‖C3-PTZ (static)	BMP-TFSI|FEC	0.5 M	1	3.40	34[a]	47[b]	100	98	97	
Li‖CP (static)	BMP-TFSI|FEC	0.5 M	1	4.09	30[a]	52[b]	100	92	92	
Li‖Tri-TEMPO (flow)	BMP-TFSI|FEC	0.5 M	2	3.56	33[a]	49[b]	100	96	85	
MV‖TEMPO	H_2O|[P44414]Cl	20 mM	0.16	1.6	13[a]		25	80	-	1
MV‖TEMPO	H_2O|PEG	0.1 M	-	1.23	2.2[a]		550	82	99.9	2
Zn‖TEMPO	H_2O|TEGDME	0.5 M	-	1.5	12.5[a]		500	99	99	3
DMFc	TFT|DCE	0.1 M	-	0.6	3.2[a]		25	80	-	4
pBQ‖H_2Q	H_2O| $Pyr_{14}TFSI$	20 mM	0.20	1.4	5.6[a]		75	80	98	5
All-iron	H_2O|ethyl acetate| $Pyr_{14}TFSI$	0.1 M	0.43, 0.08	1.5	2.9[a]		25	80	60	6
2,3-DMAQ‖H_2Q	H_2O|$Pyr_{14}TFSI$	20 mM	1.0	1.9	3.9[a]		25	80	~85	7
OilBlue N‖H_2Q	H_2O|$Pyr_{14}TFSI$	20 mM	1.0	2.1	4.1[a]		25	90	~90	7
pBQ‖H_2Q	H_2O|$Pyr_{14}TFSI$	20 mM	1.0	1.0	2.8[a]		14	40	~60	7
pBQ‖TEMPO	H_2O|$Pyr_{14}TFSI$	40 mM	1.0	1.5	3.5[a]		25	80	~65	7
pBQ‖OH-TEMPO	H_2O|$Pyr_{14}TFSI$	0.1 M	1.0	1.0	1.9[a]		300	80	~85	7
Zn‖PTZ	CH_2Cl_2|H_2O	0.5 M	-	1.67	13.6[a]		202	96	80	8
Zn‖PTZ	PC|MeCN	0.5 M	8.54	1.65	12.8[a]		194	99	~95	9
Li‖2-EAQ	TEGDME/NFTOS	0.2 M	0.05	2.22	21[a]		100	-	-	10

RMs: redox materials, [a]demonstrated experimental energy density, [b]theoretical energy density.

Supplementary Table 5. Unit price cost of materials components used in this study.

	**Materials**	**Unit cost ($/Kg)**	**Amount used**	**Price ($)**	**Supplementary References**
	FEC	90			12
	LiTFSI	180			12
	BMP-TFSI	300			13
	$LiClO_4$	45			-
One-step synthesis	Cost estimation for **C3-PTZ**				
	KOH	0.55	2.2 g	0.00121	14
	DMSO	1.63	60 mL	0.0098	15
	PTZ	72	5.2 g	0.518	16
	C_3H_7I	10	3.1 mL	0.031	-
			Total cost for 4.2 g of **C3-PTZ**	~0.56 (**C3-PTZ unit cost= 133** $/Kg)	
Two-step synthesis	Cost estimation for **Tri-TEMPO**				
Step-1	$C_3Cl_3N_3$	56	37 g	2.08	17
	2,2,6,6-tetramethylpiperidine	86	94 g	8.08	12
	Acetone	0.76	230 mL	0.175	18
			Total cost for 106 g of product from reaction 1	10.33 (or ~95 $/Kg)	-
Step-2	Reaction 1 product	95	27 g	2.56	-
	Ethanol	0.57	150 mL	0.0855	19
	NaOH	0.35	24 g	0.0084	20
	$Mg(OH)_2$	4.7	0.2 g	0.00094	-
	MnO_2	2.85	0.1 g	0.000185	-
	H_2O_2	1.84	26 g	0.047	21
			Total cost for 29.41 g of **Tri-TEMPO**	2.703 (**Tri-TEMPO** unit cost = **91** $/Kg)	
Two-step reaction	Cost estimation for **CP**				
Step-1	Sodium trichloroacetate	20	300 g	6	22

	Trichloroethylene	1.2	500 mL	0.6	23
	1 2-dimethoxyethane	3.5	145 mL	0.508	24
	Total cost for 60 g of product from reaction 1			6.57 (110 $/kg)	-
Step-2	Reaction 1 product	110	0.430 g	0.047	-
	N-Ethylbutylamine	2.8	1.4 mL	0.004	-
	Ammonium hexafluorophosphate	270	0.7 g	0.189	25
	Total cost for 0.694 g of **CP**			0.24 (**CP** unit cost = **346** $/Kg)	
Cost of FEC				22.5 ($/L)	
Cost of ionic liquid				46 ($/L)	
Cost of graphite felt and Li metal				4 ($/cm^2)	

Supplementary Note 1

Economic analysis

Analyzing the cost or conducting an economic assessment of membrane-free batteries is essential. The cost of the Tri-TEMPO, C3-PTZ, and CP nonaqueous membrane-free batteries is primarily determined by the price of the components used in both the catholyte and anolyte layers, including redox-active materials, FEC, $LiClO_4$, GF, BMP-TFSI, LiTFSI, and Li metal. The estimated costs of each component are detailed in Supplementary Table 5. The costs of the components used in this study are relatively comparable to those of the Li||TEMPO membrane-based nonaqueous battery[11]. In contrast, the major expenses associated with nonaqueous Li redox flow batteries stem from typical components including the bipolar plate, GF, membrane, and current collector plate. These components account for approximately 18%, 28%, 35%, and 3% of the total cost, respectively. Despite the higher costs of the ILs used in this study and the synthesized compounds, the elimination of high-cost membrane separators significantly improves the cost-effectiveness of the developed system. Therefore, the proposed batteries are comparatively cost-effective.

Supplementary References List

(1) Navalpotro, P., Neves, C. M. S. S., Palma, J., Freire, M. G., Coutinho, J. A. P., Marcilla, R. Pioneering Use of Ionic Liquid-Based Aqueous Biphasic Systems as Membrane-Free Batteries. *Adv. Sci.* **5**, 1800576 (2018).

(2) Navalpotro, P., Trujillo, C., Montes, I., Neves, C. M. S. S., Palma, J., Freire, M. G., Coutinho, J. A. P., Marcilla, R. Critical Aspects of Membrane-Free Aqueous Battery Based on Two Immiscible Neutral Electrolytes. *Energy Storage Mater.* **26**, 400–407 (2020). https://doi.org/10.1016/j.ensm.2019.11.011.

(3) Meng, J., Tang, Q., Zhou, L., Zhao, C., Chen, M., Shen, Y., Zhou, J., Feng, G., Shen, Y., Huang, Y. A Stirred Self-Stratified Battery for Large-Scale Energy Storage. *Joule* **4**, 953–966 (2020).

(4) Peljo, P., Bichon, M., Girault, H. H. Ion Transfer Battery: Storing Energy by Transferring Ions across Liquid–Liquid Interfaces. *Chem. Commun.* **52**, 9761–9764 (2016).

(5) Navalpotro, P., Palma, J., Anderson, M., Marcilla, R. A Membrane-Free Redox Flow Battery with Two Immiscible Redox Electrolytes. *Angew. Chem. Int. Ed.* **129**, 12634-12639 (2017). https://doi.org/10.1002/anie.201704318.

(6) Bamgbopa, M. O., Shao-Horn, Y., Hashaikeh, R., Almheiri, S. Cyclable Membraneless Redox Flow Batteries Based on Immiscible Liquid Electrolytes: Demonstration with All-Iron Redox Chemistry. *Electrochim. Acta* **267**, 41–50 (2018).

(7) Navalpotro, P., Sierra, N., Trujillo, C., Montes, I., Palma, J., Marcilla, R. Exploring the Versatility of Membrane-Free Battery Concept Using Different Combinations of Immiscible Redox Electrolytes. *ACS Appl. Mater. Interfaces* **10**, 41246–41256 (2018).

(8) Chai, J., Lashgari, A., Eisenhart, A. E., Wang, X., Beck, T. L., Jiang, J. Biphasic, Membrane-Free Zn/Phenothiazine Battery: Effects of Hydrophobicity of Redox Materials on Cyclability. *ACS Mater. Lett.* **3**, 337–343 (2021).

(9) Wang, X., Lashgari, A., Chai, J., Jiang, J. A Membrane-Free, Aqueous/Nonaqueous Hybrid Redox Flow Battery. *Energy Storage Mater.* **45**, 1100–1108 (2022).

(10) Liu, X., Song, X., Guo, Z., Bian, T., Zhang, J., Zhao, Y. Biphasic Electrolyte Inhibiting the Shuttle Effect of Redox Molecules in Lithium-Metal Batteries. *Angew. Chem. Int. Ed.* **60** 16360–16365 (2021).

(11) Li, Z., Fang, X., Cheng, L., Wei, X., Zhang, L. Techno-Economic Analysis of Non-Aqueous Hybrid Redox Flow Batteries. *J. Power Sources* **536**, 231493 (2022).

(12) Li, Z.; Fang, X.; Cheng, L.; Wei, X.; Zhang, L. Techno-Economic Analysis of Non-Aqueous Hybrid Redox Flow Batteries. *J. Power Sources* **2022**, *536*, 231493. https://doi.org/10.1016/j.jpowsour.2022.231493.

(13) https://cen.acs.org/materials/ionic-liquids/time-ionic-liquids/98/i5 (Last accessed: October 2022)

(14) https://www.globenewswire.com/en/news-release/2022/06/09/2459386/0/en/EU-Potassium-Hydroxide-Market-Report-Suppliers-Buyers-Prices-and-Forecast-IndexBox.html. (Last accessed: October 2022)

(15) https://www.pharmacmpass.com/price/dimethyl-sulfoxide. (Last accessed: October 2022).

(16) https://www.tcichemicals.com/US/en/p/P0106 (Last accessed: October 2022)

(17) https://www.procurementresource.com/resource-center/cyanuric-chloride-price-trends. (Last accessed: October 2022).

(18) https://www.chemanalyst.com/Pricing-data/acetone-12#:~:text=Acetone%20prices%20settled%20at%20USD%20761%20per%20MT%20in%20January%202022. (Last accessed: October 2022).
(19) https://tradingeconomics.com/commodity/ethanol. (Last accessed: October 2022).
(S20) https://www.indexbox.io/blog/caustic-soda-price-per-ton-april 2022/#:~:text=U.S.%20Caustic%20Sode%20Price%20per,monthly%20rate%20of%20%2B4.8%25. (Last accessed: October 2022).
(21) https://www.chemanalyst.com/Pricing-data/hydrogen-peroxide-1169. (Last accessed: October 2022).
(22) https://www.ottokemi.com/sodium-compounds/sodium-trichl198.aspx. (Last accessed: October 2022).
(23) https://www.indexbox.io/store/world-trichloroethylene-and-tetrachloroethylene perchloroethylene-market-analysis-forecast-size-trends-and-insights/. (Last accessed: October 2022).
(24) https://www.alibaba.com/showroom/1-2-dimethoxyethane.html. (Last accessed: October 2022).
(25) https://www.indiamart.com/proddetail/ammonium-hexafluorophosphate-nh4pf6-cas-16941-11-0-25402566797.html. (Last accessed: October 2022).
